# Supplementary figures and images for: Bacillus velezensis HBXN2020 alleviates Salmonella Typhimurium infection in mice by improving intestinal barrier integrity and reducing inflammation
Source: eLife. 2024 Nov 19;13:RP93423. doi: 10.7554/eLife.93423 (PMC11575897; doi:10.7554/eLife.93423)

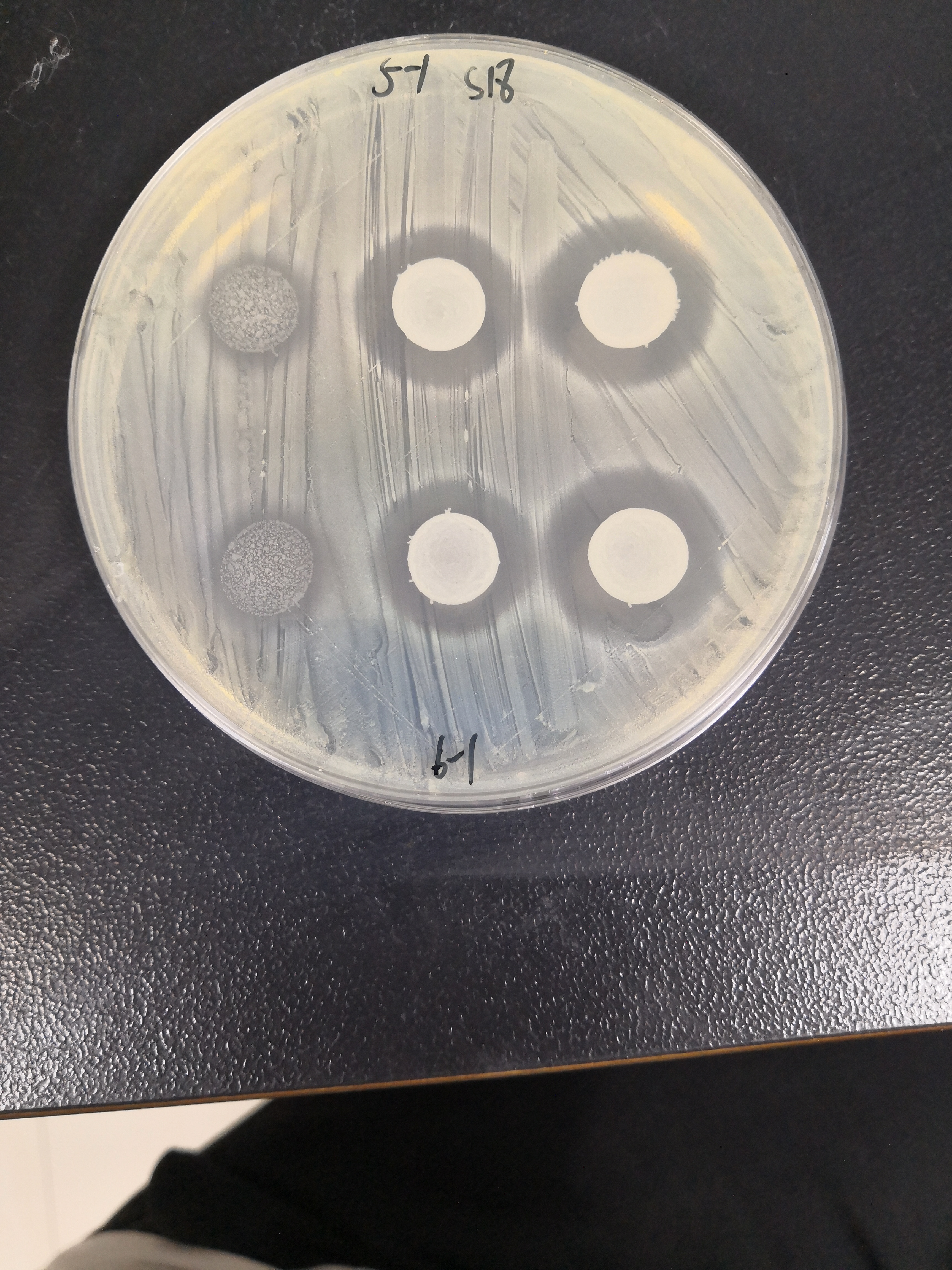

Supplement: Figure 1—figure supplement 1—source data 2. [file elife-93423-fig1-figsupp1-data2.zip › Figure 1—figure supplement 1—source data 2/Figure 1—figure supplement 1—source data 2/A.jpg]

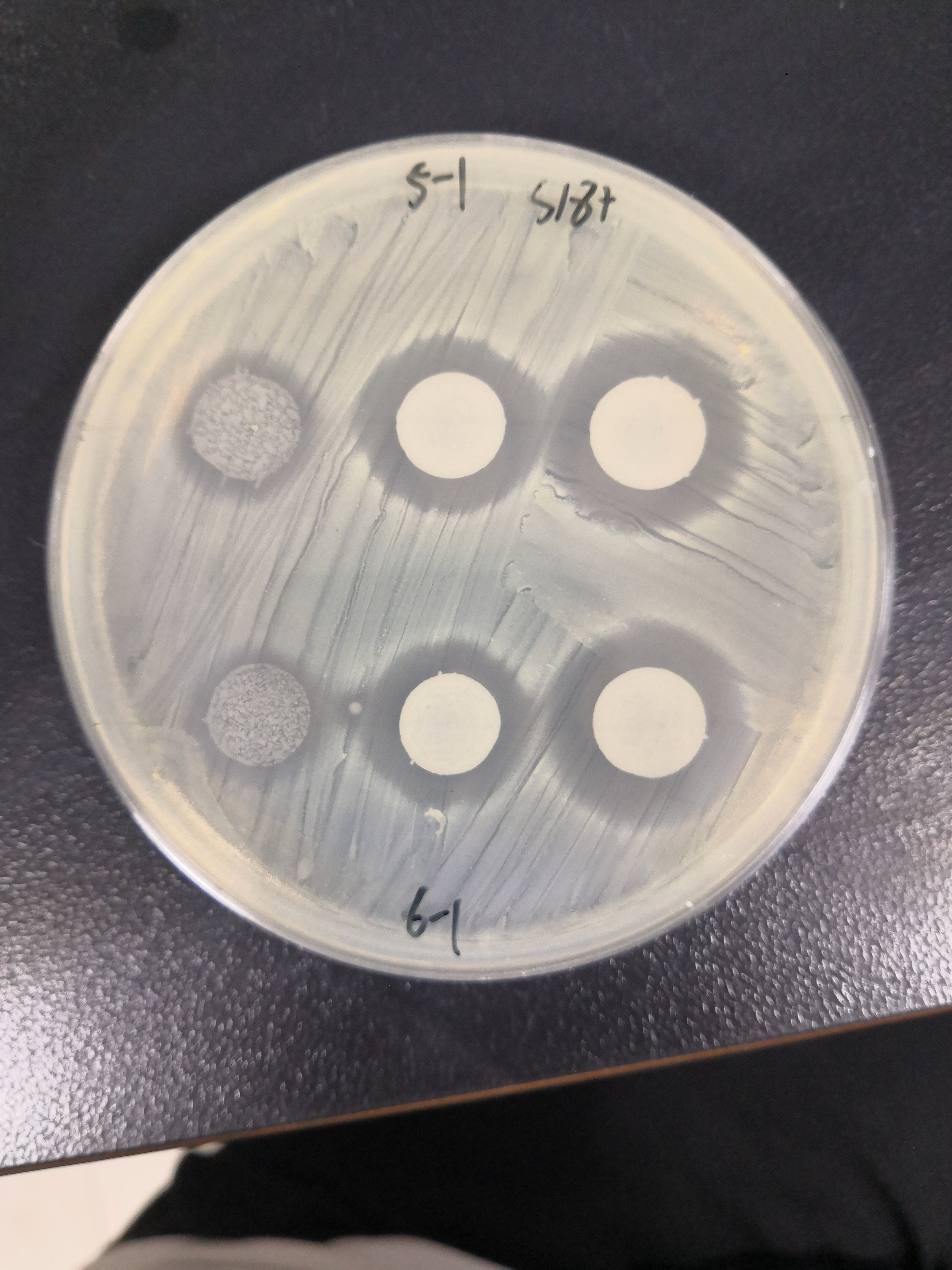

Supplement: Figure 1—figure supplement 1—source data 2. [file elife-93423-fig1-figsupp1-data2.zip › Figure 1—figure supplement 1—source data 2/Figure 1—figure supplement 1—source data 2/B.jpg]

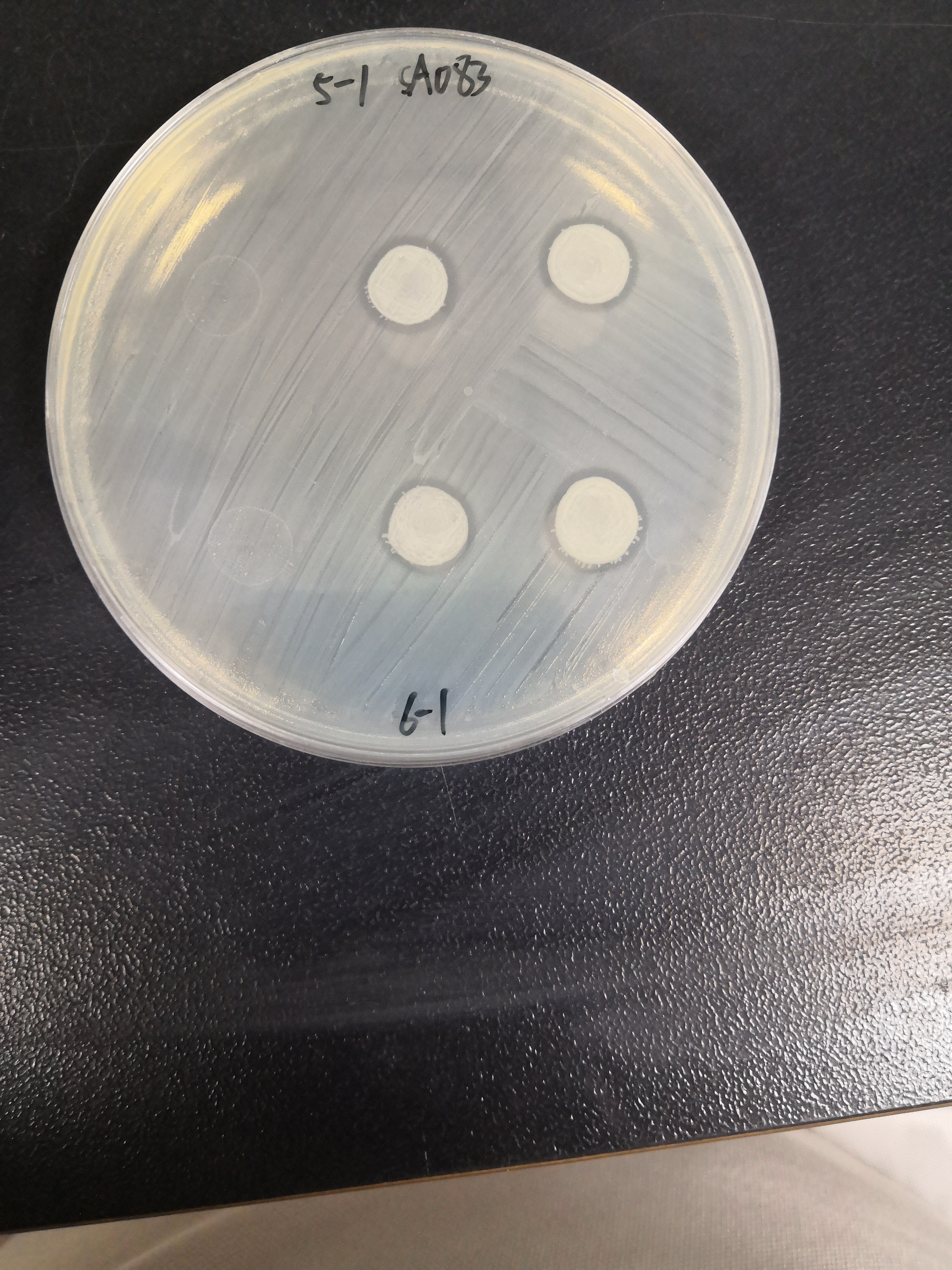

Supplement: Figure 1—figure supplement 1—source data 2. [file elife-93423-fig1-figsupp1-data2.zip › Figure 1—figure supplement 1—source data 2/Figure 1—figure supplement 1—source data 2/C.jpg]

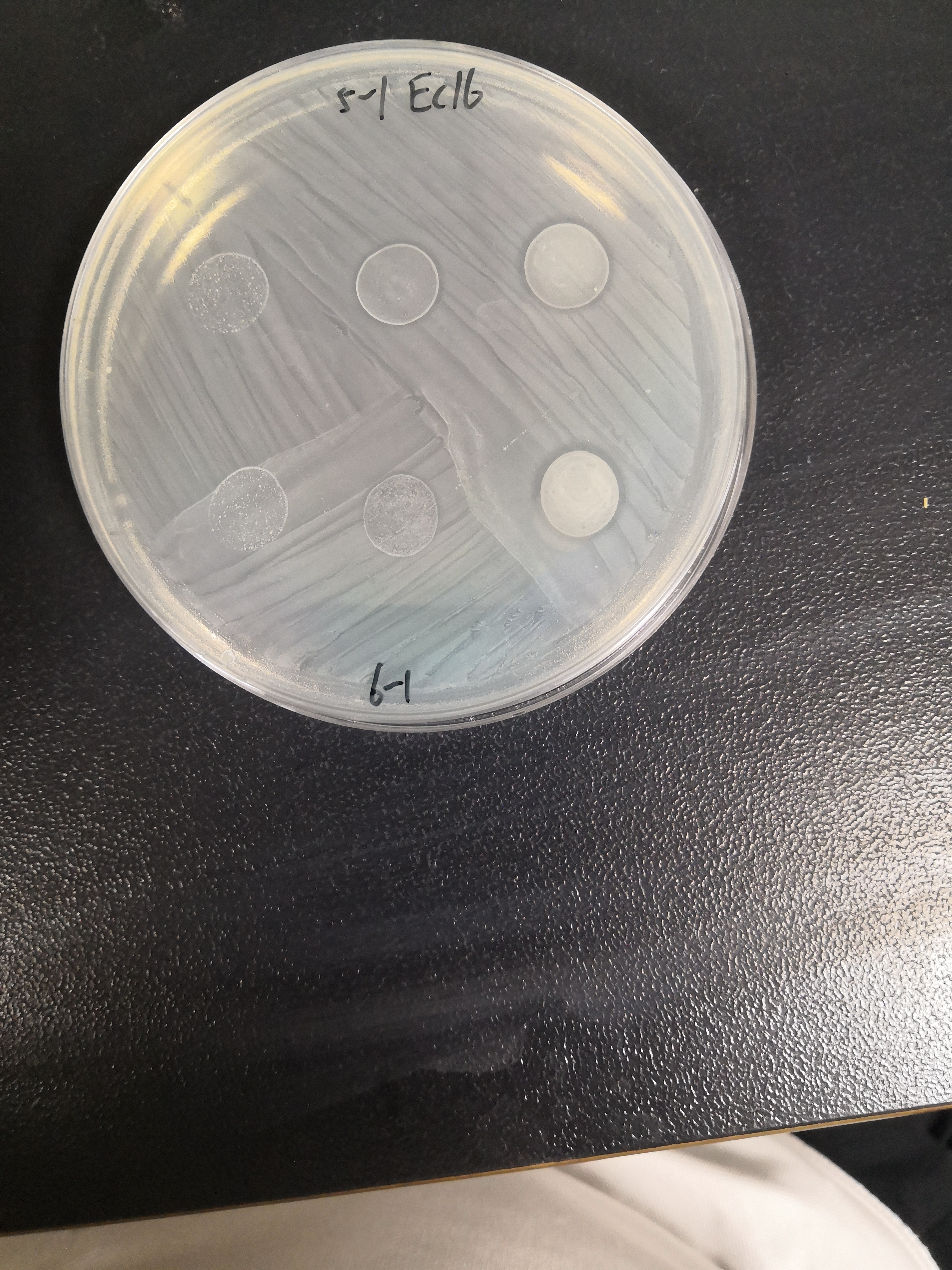

Supplement: Figure 1—figure supplement 1—source data 2. [file elife-93423-fig1-figsupp1-data2.zip › Figure 1—figure supplement 1—source data 2/Figure 1—figure supplement 1—source data 2/D.jpg]

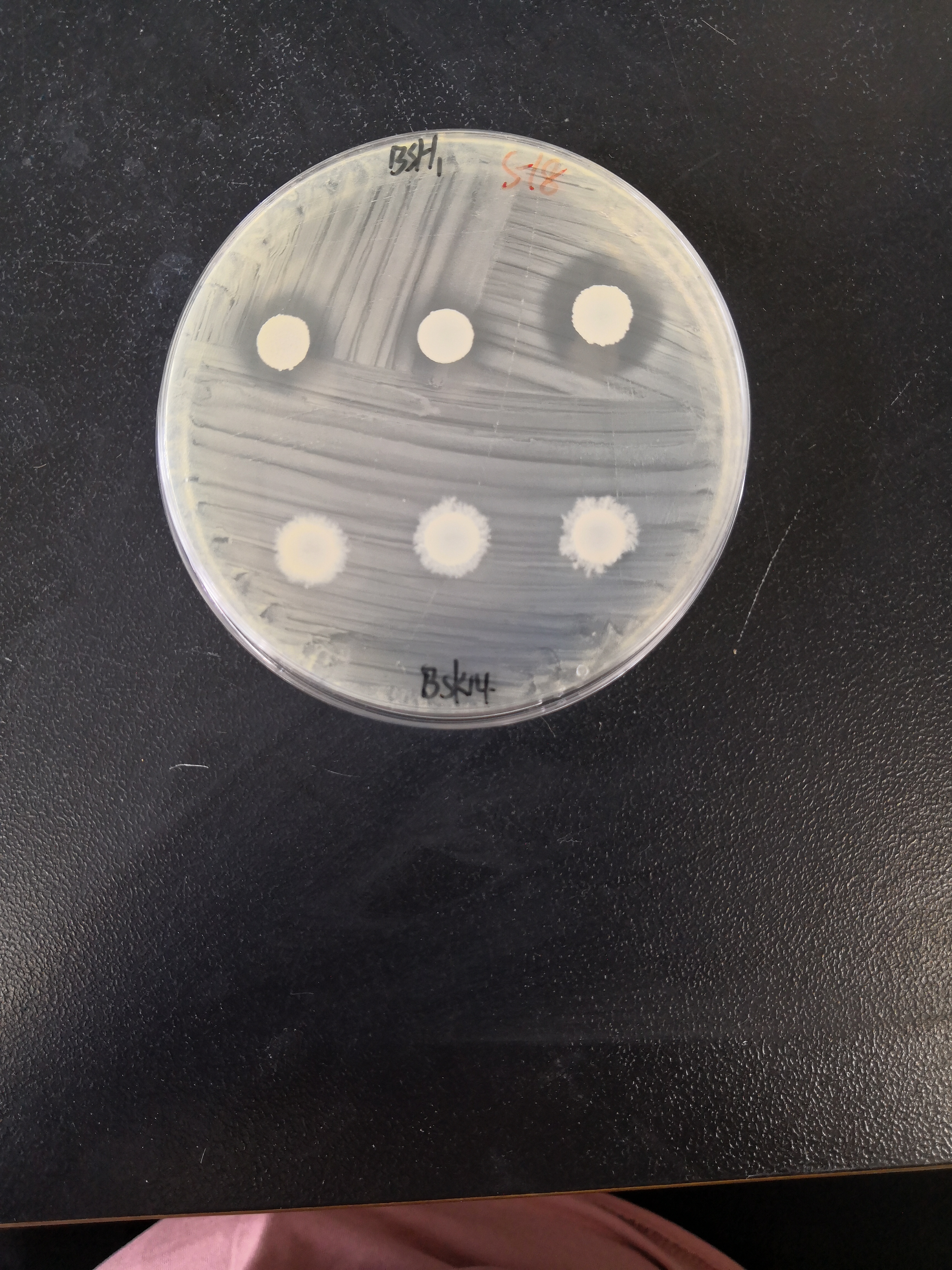

Supplement: Figure 1—figure supplement 1—source data 2. [file elife-93423-fig1-figsupp1-data2.zip › Figure 1—figure supplement 1—source data 2/Figure 1—figure supplement 1—source data 2/E.jpg]

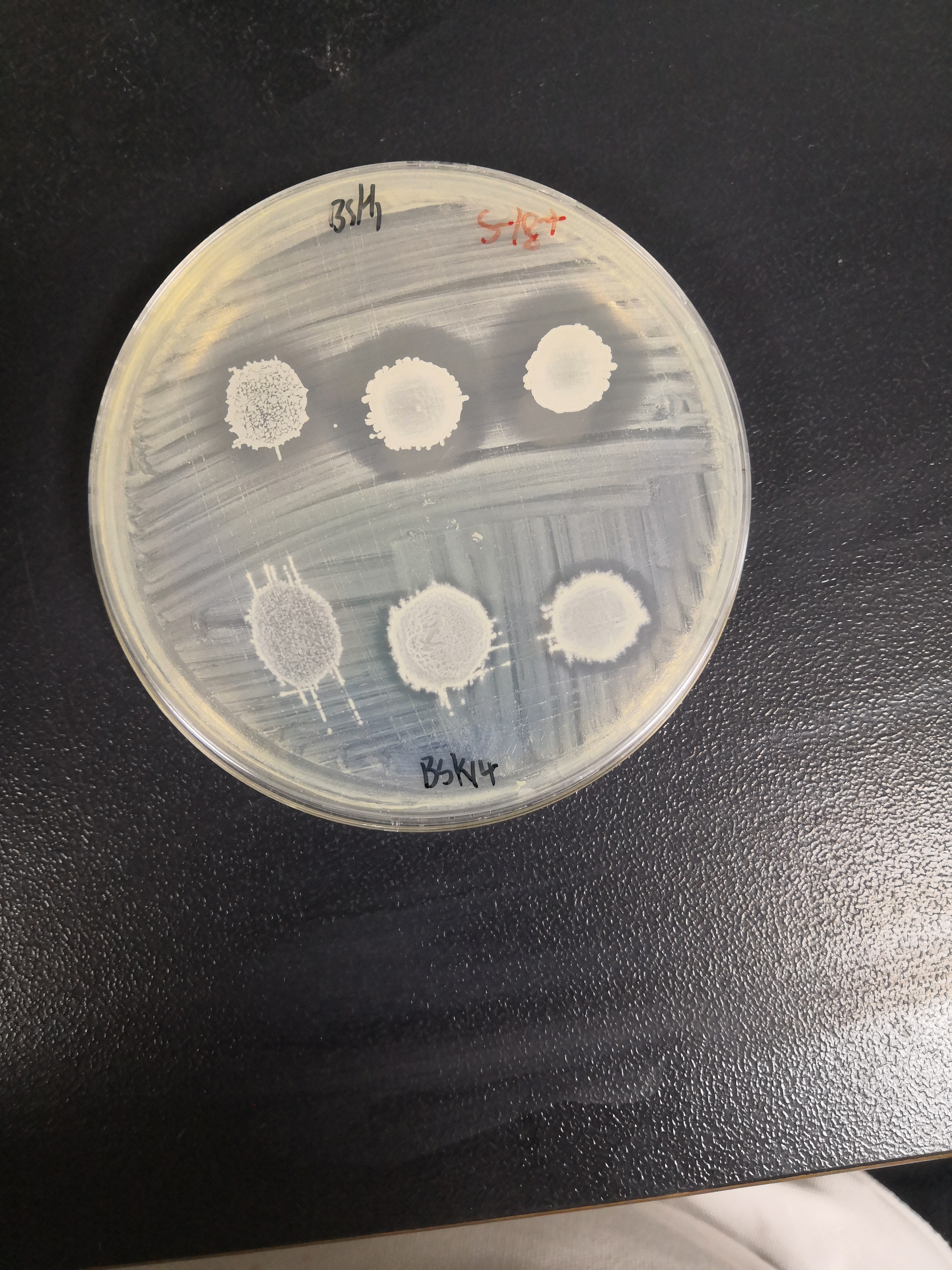

Supplement: Figure 1—figure supplement 1—source data 2. [file elife-93423-fig1-figsupp1-data2.zip › Figure 1—figure supplement 1—source data 2/Figure 1—figure supplement 1—source data 2/F.jpg]

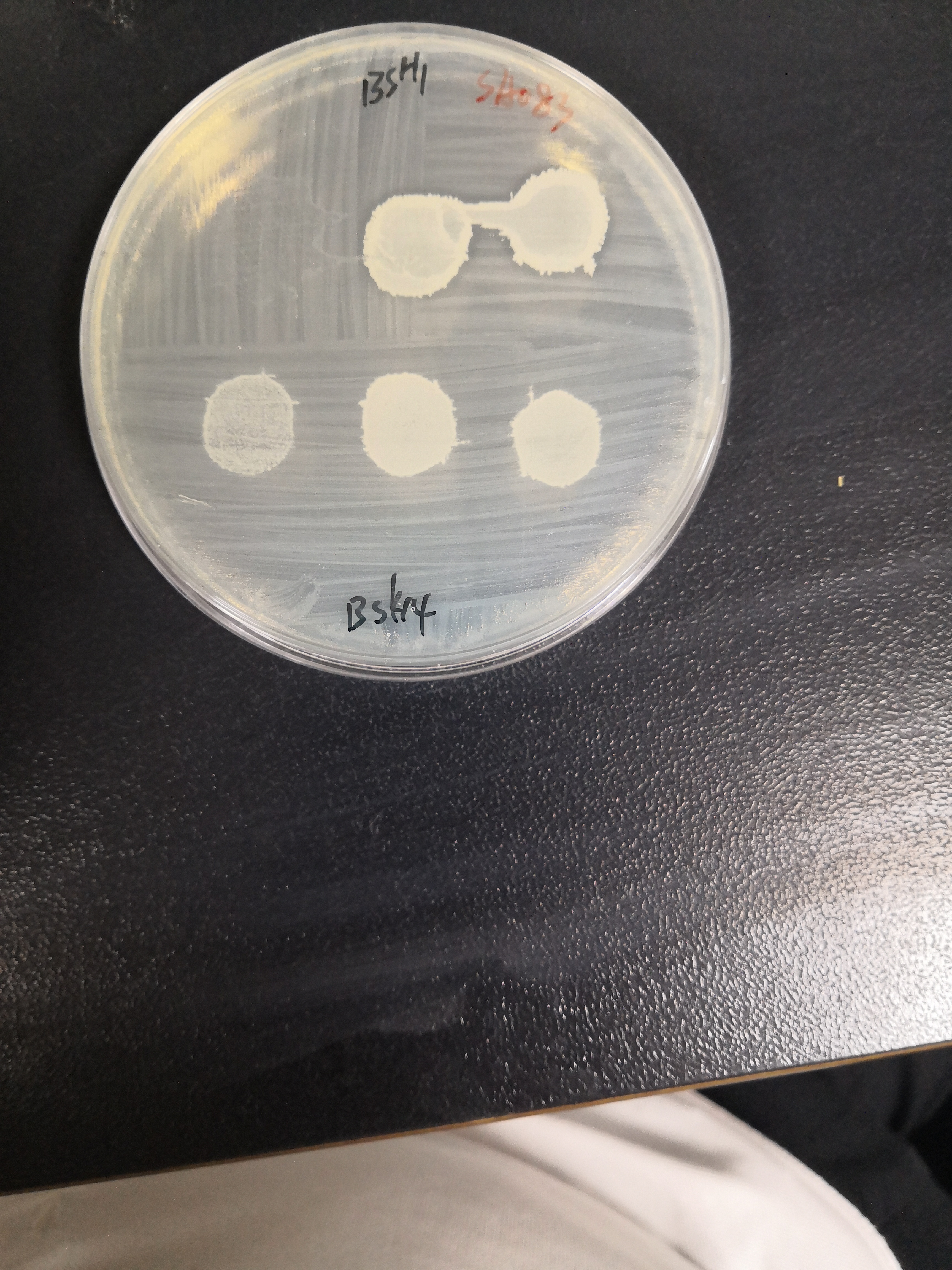

Supplement: Figure 1—figure supplement 1—source data 2. [file elife-93423-fig1-figsupp1-data2.zip › Figure 1—figure supplement 1—source data 2/Figure 1—figure supplement 1—source data 2/G.jpg]

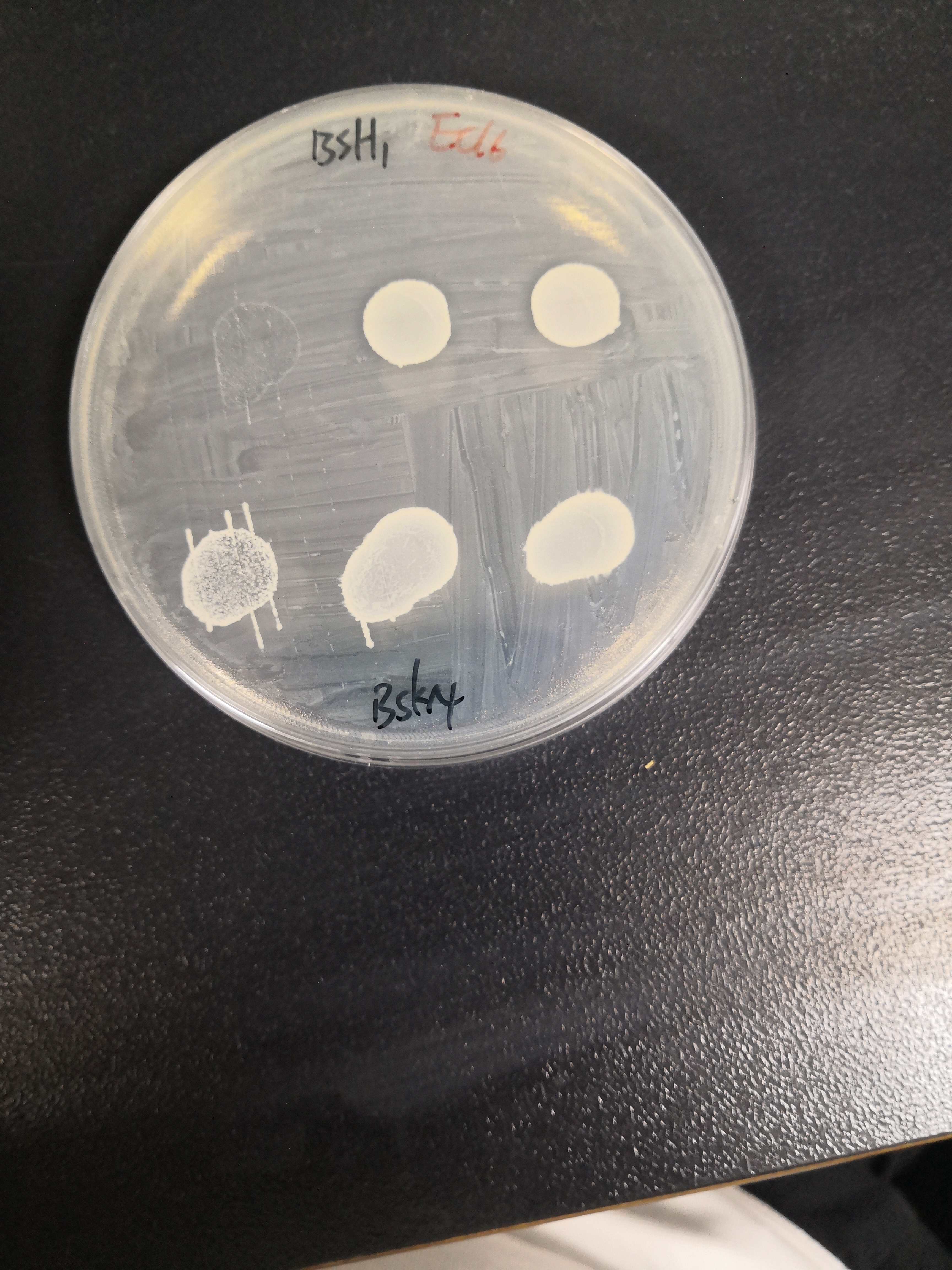

Supplement: Figure 1—figure supplement 1—source data 2. [file elife-93423-fig1-figsupp1-data2.zip › Figure 1—figure supplement 1—source data 2/Figure 1—figure supplement 1—source data 2/H.jpg]

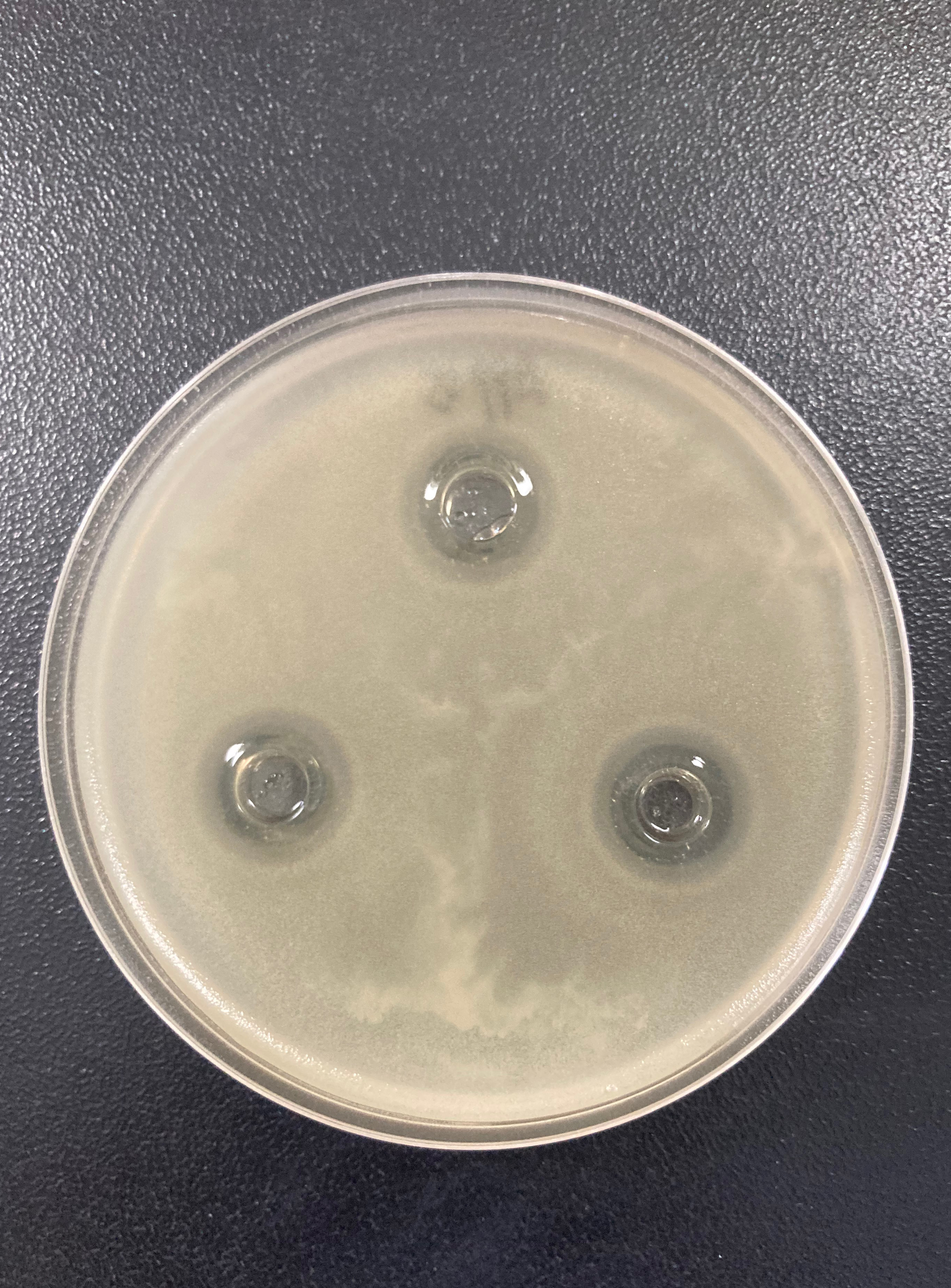

Supplement: Figure 2—figure supplement 1—source data 2. [file elife-93423-fig2-figsupp1-data2.zip › Figure 2—figure supplement 1—source data 2/Figure 2—figure supplement 1—source data 2/A. pleuropneumoniae APP015.jpg]

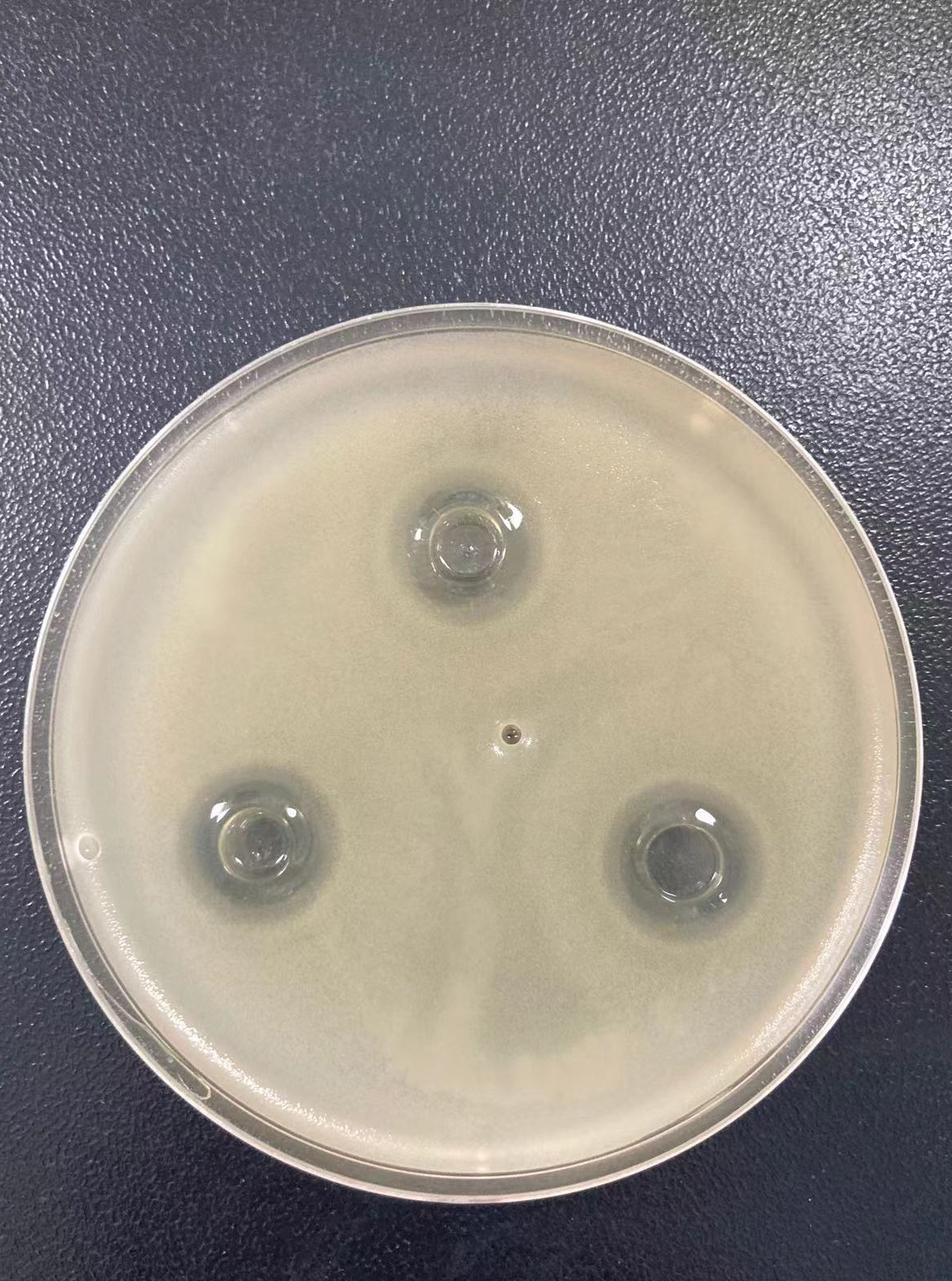

Supplement: Figure 2—figure supplement 1—source data 2. [file elife-93423-fig2-figsupp1-data2.zip › Figure 2—figure supplement 1—source data 2/Figure 2—figure supplement 1—source data 2/A. pleuropneumoniae APP017.jpg]

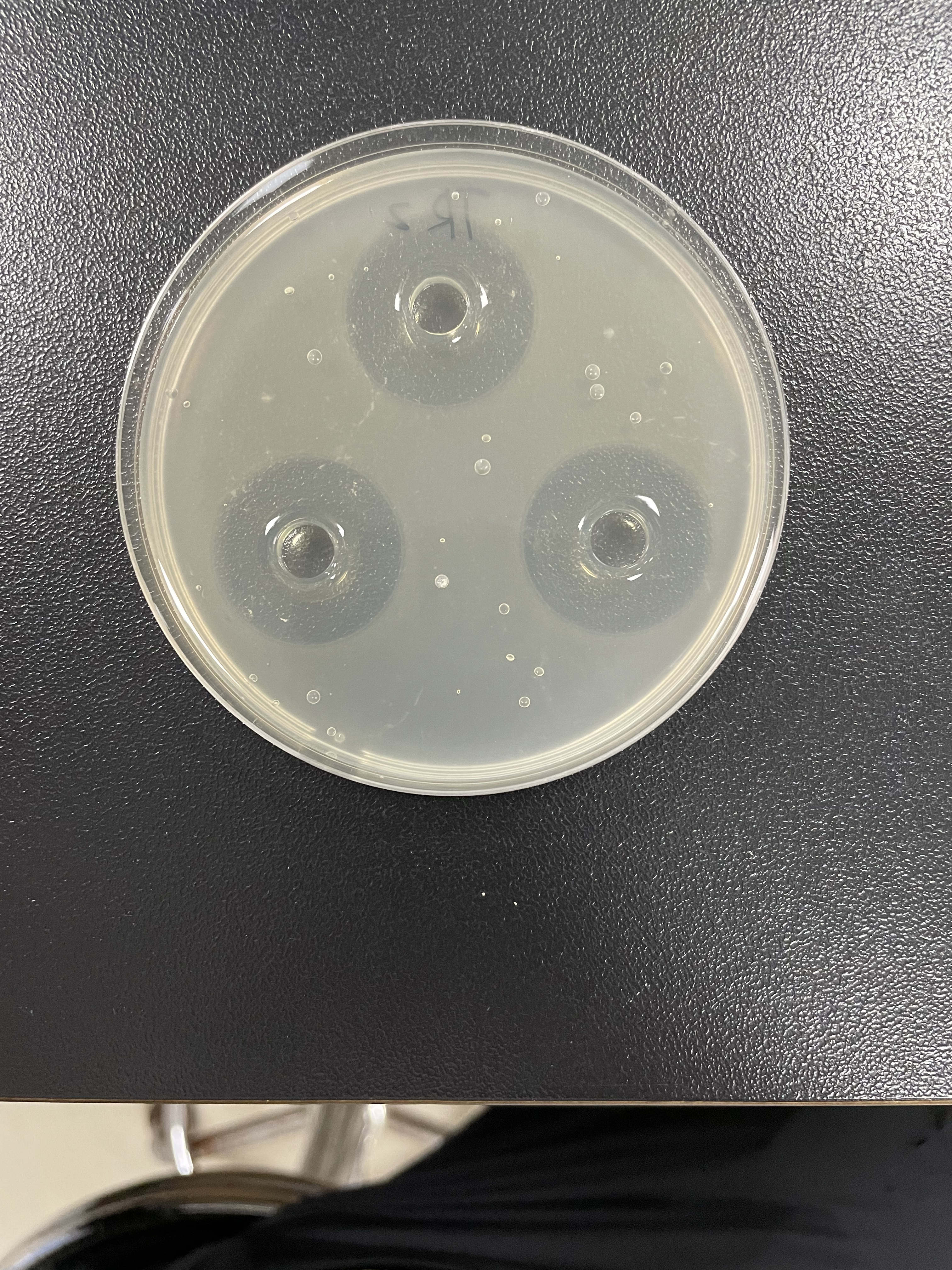

Supplement: Figure 2—figure supplement 1—source data 2. [file elife-93423-fig2-figsupp1-data2.zip › Figure 2—figure supplement 1—source data 2/Figure 2—figure supplement 1—source data 2/C. perfringens CP002.jpg]

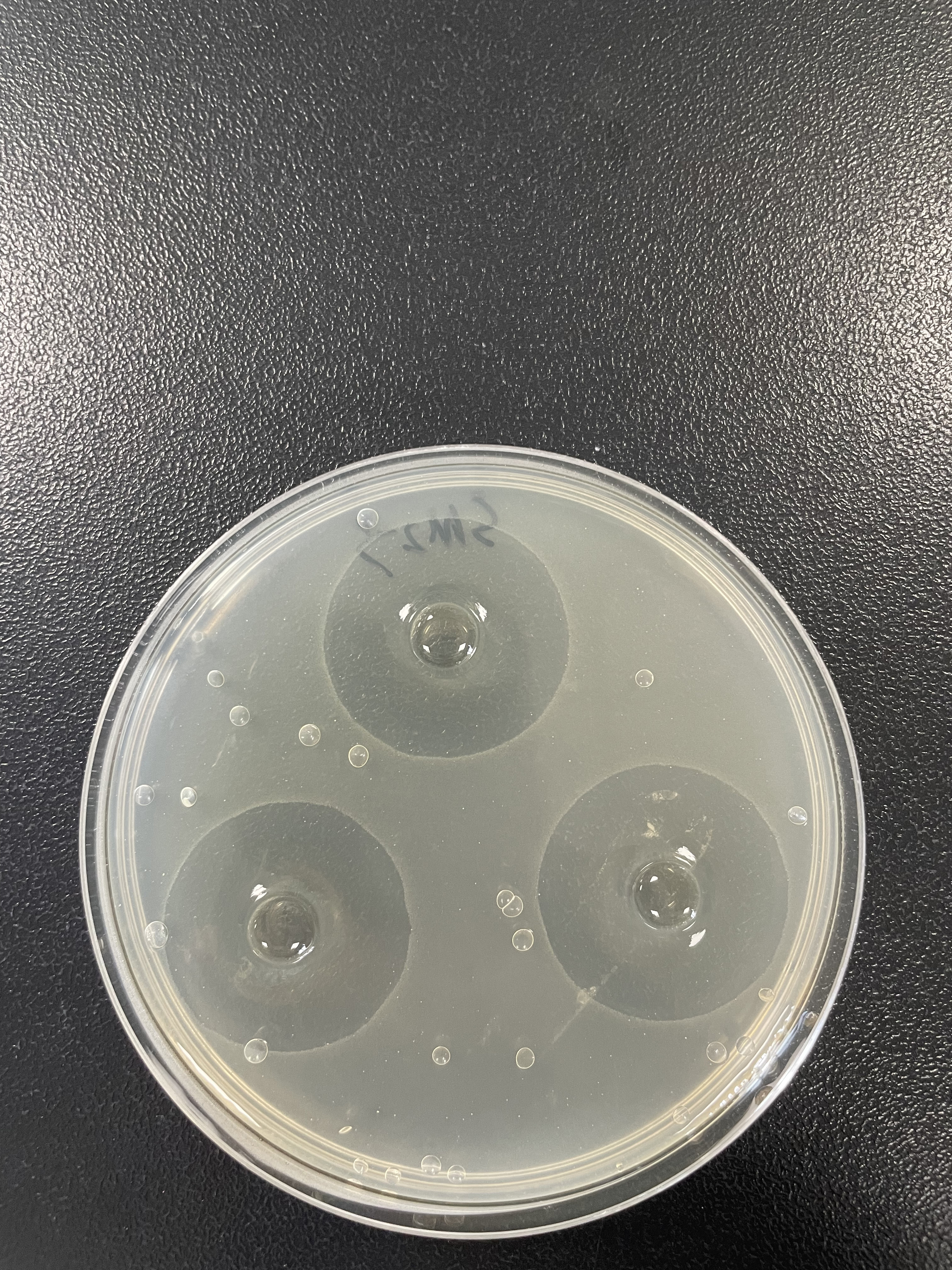

Supplement: Figure 2—figure supplement 1—source data 2. [file elife-93423-fig2-figsupp1-data2.zip › Figure 2—figure supplement 1—source data 2/Figure 2—figure supplement 1—source data 2/C. perfringens CP023.jpg]

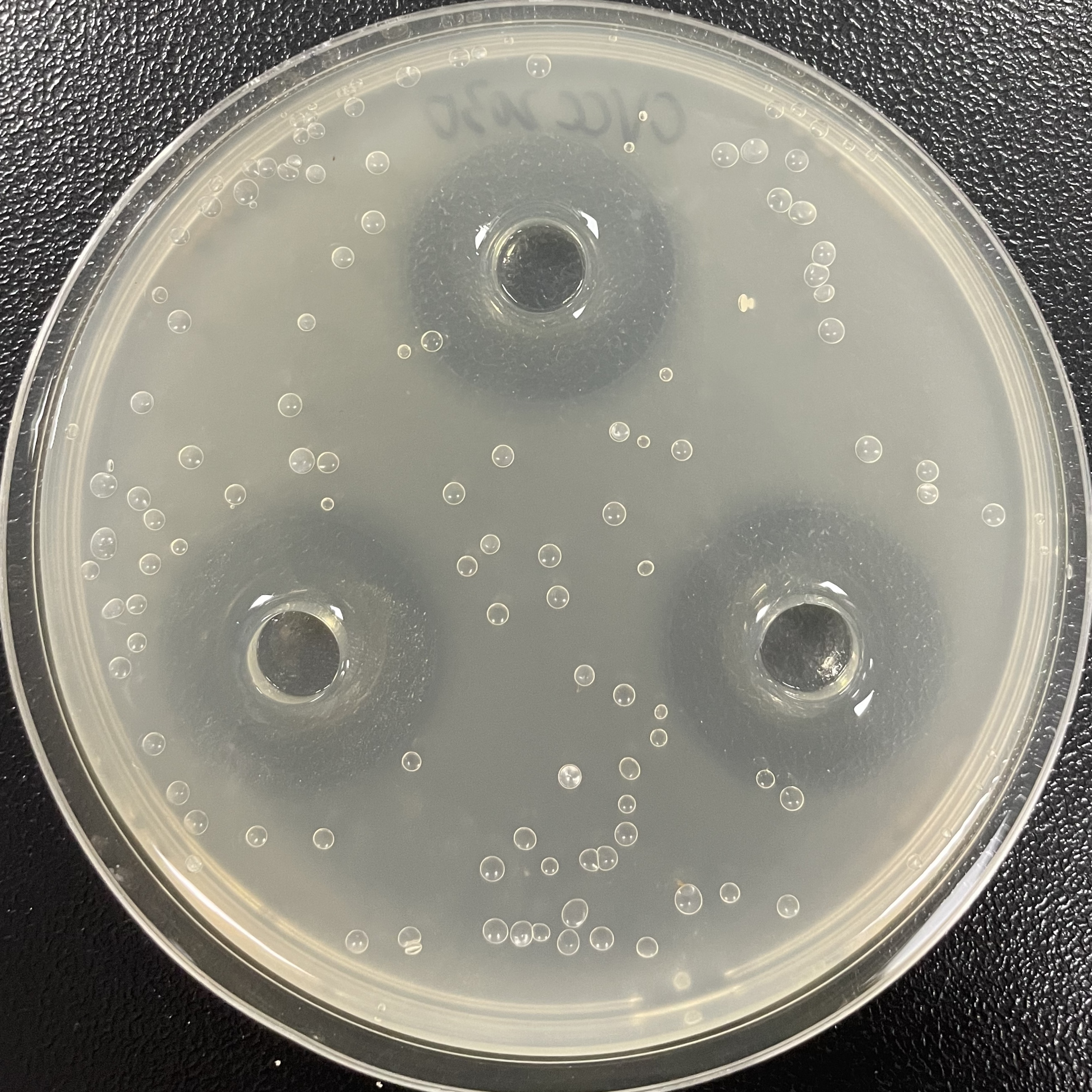

Supplement: Figure 2—figure supplement 1—source data 2. [file elife-93423-fig2-figsupp1-data2.zip › Figure 2—figure supplement 1—source data 2/Figure 2—figure supplement 1—source data 2/C. perfringens CVCC2030.jpg]

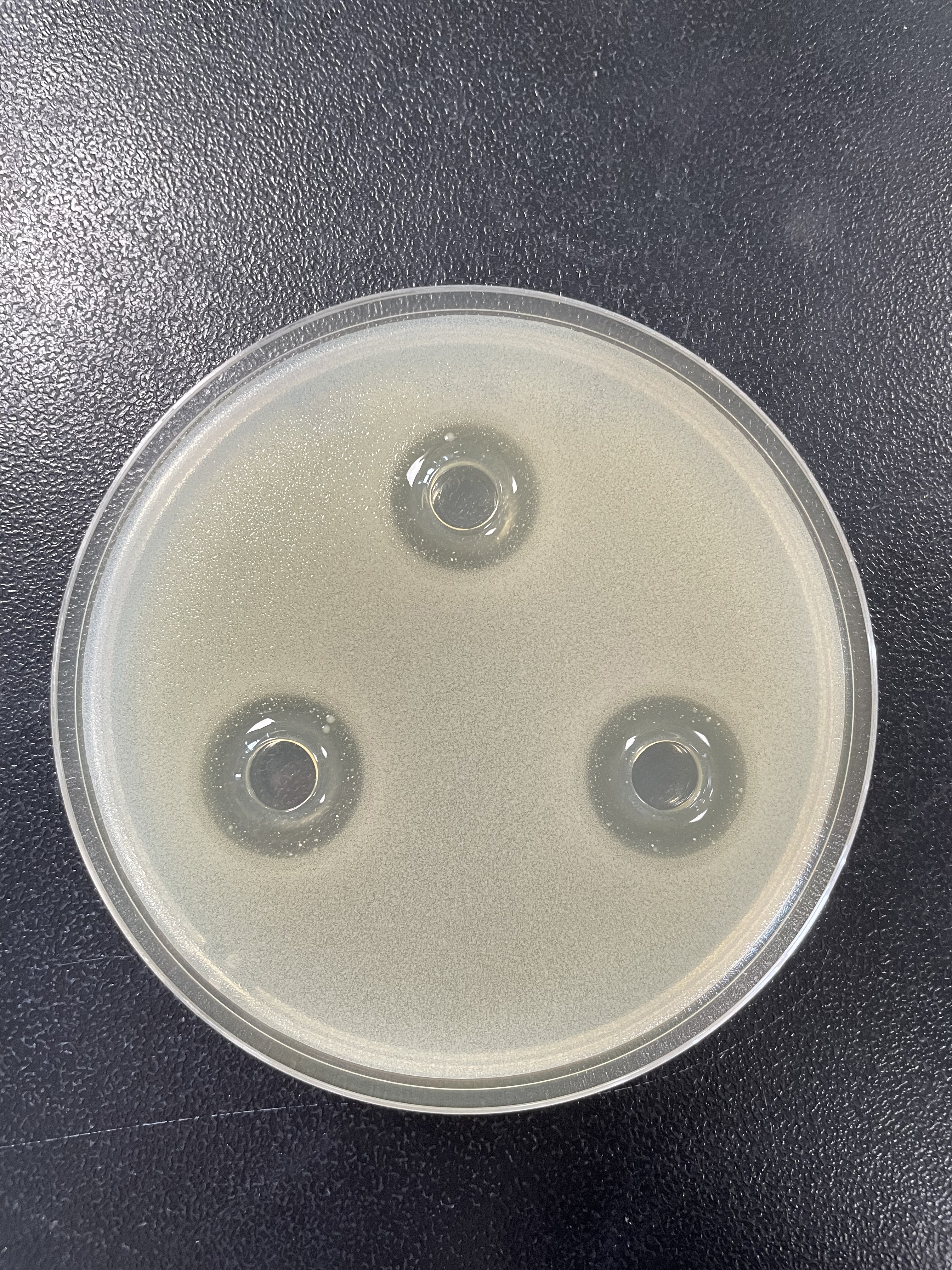

Supplement: Figure 2—figure supplement 1—source data 2. [file elife-93423-fig2-figsupp1-data2.zip › Figure 2—figure supplement 1—source data 2/Figure 2—figure supplement 1—source data 2/E. coli ATCC25922.jpg]

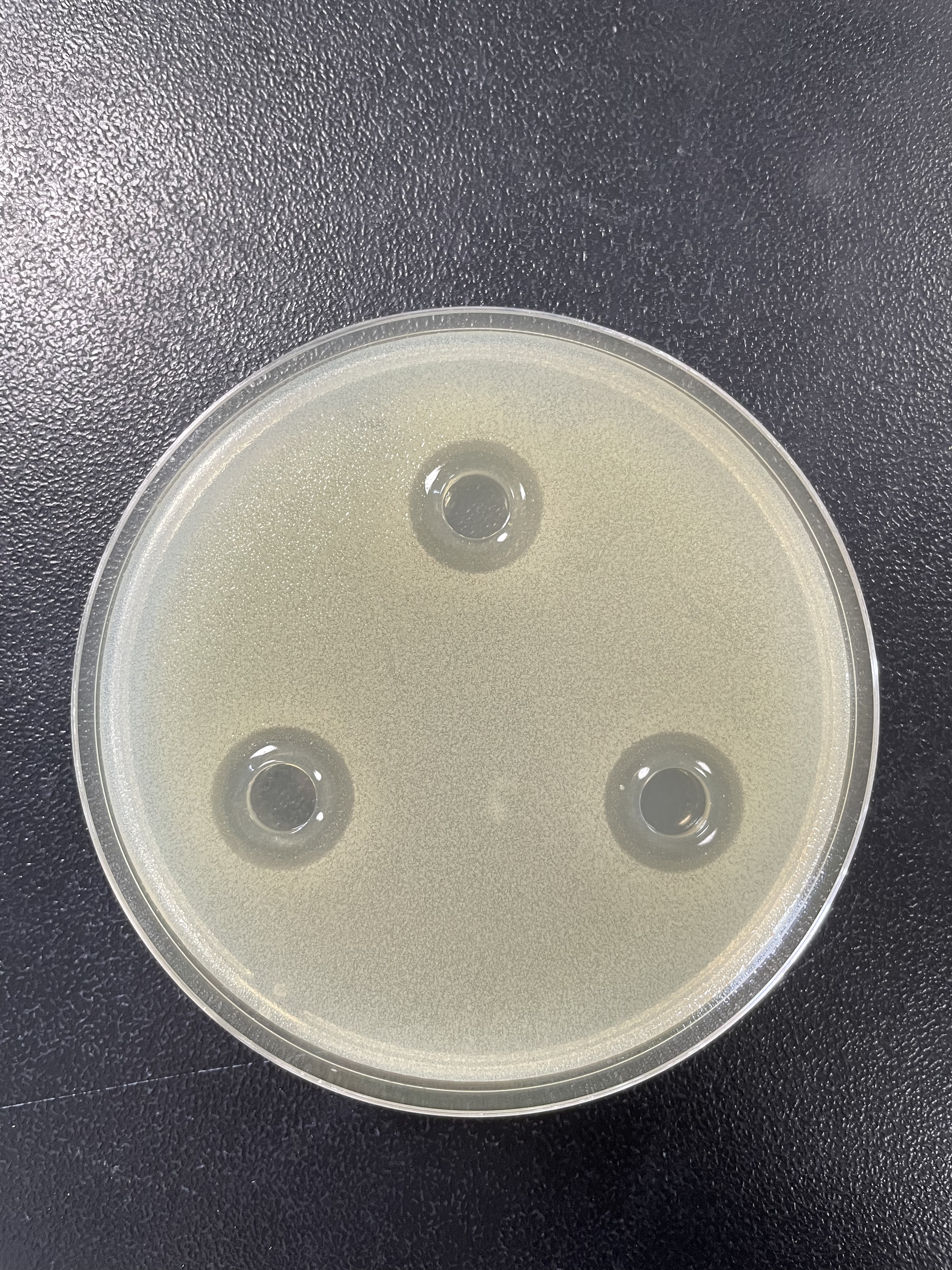

Supplement: Figure 2—figure supplement 1—source data 2. [file elife-93423-fig2-figsupp1-data2.zip › Figure 2—figure supplement 1—source data 2/Figure 2—figure supplement 1—source data 2/E. coli ATCC35150.jpg]

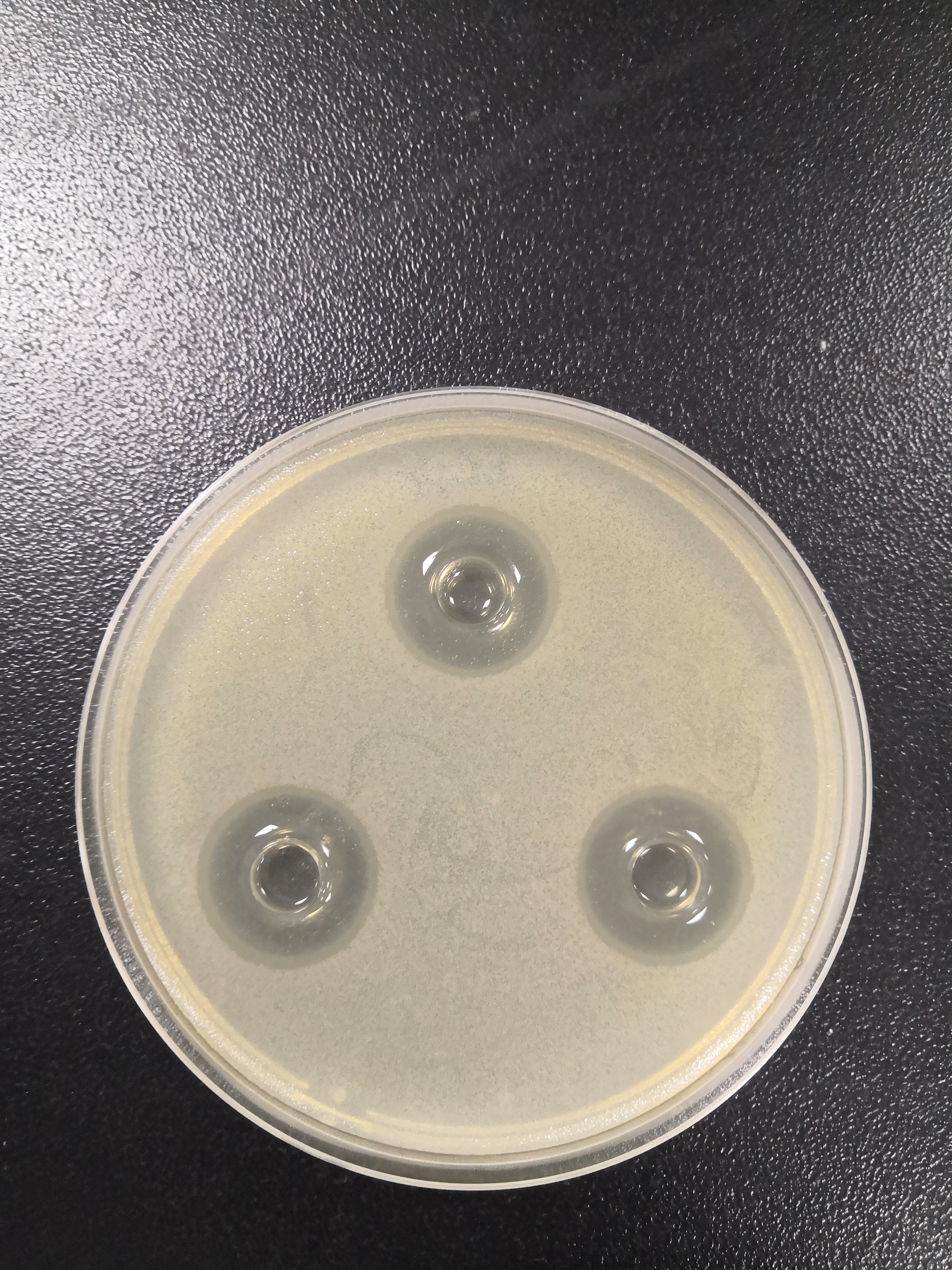

Supplement: Figure 2—figure supplement 1—source data 2. [file elife-93423-fig2-figsupp1-data2.zip › Figure 2—figure supplement 1—source data 2/Figure 2—figure supplement 1—source data 2/E. coli EC024.jpg]

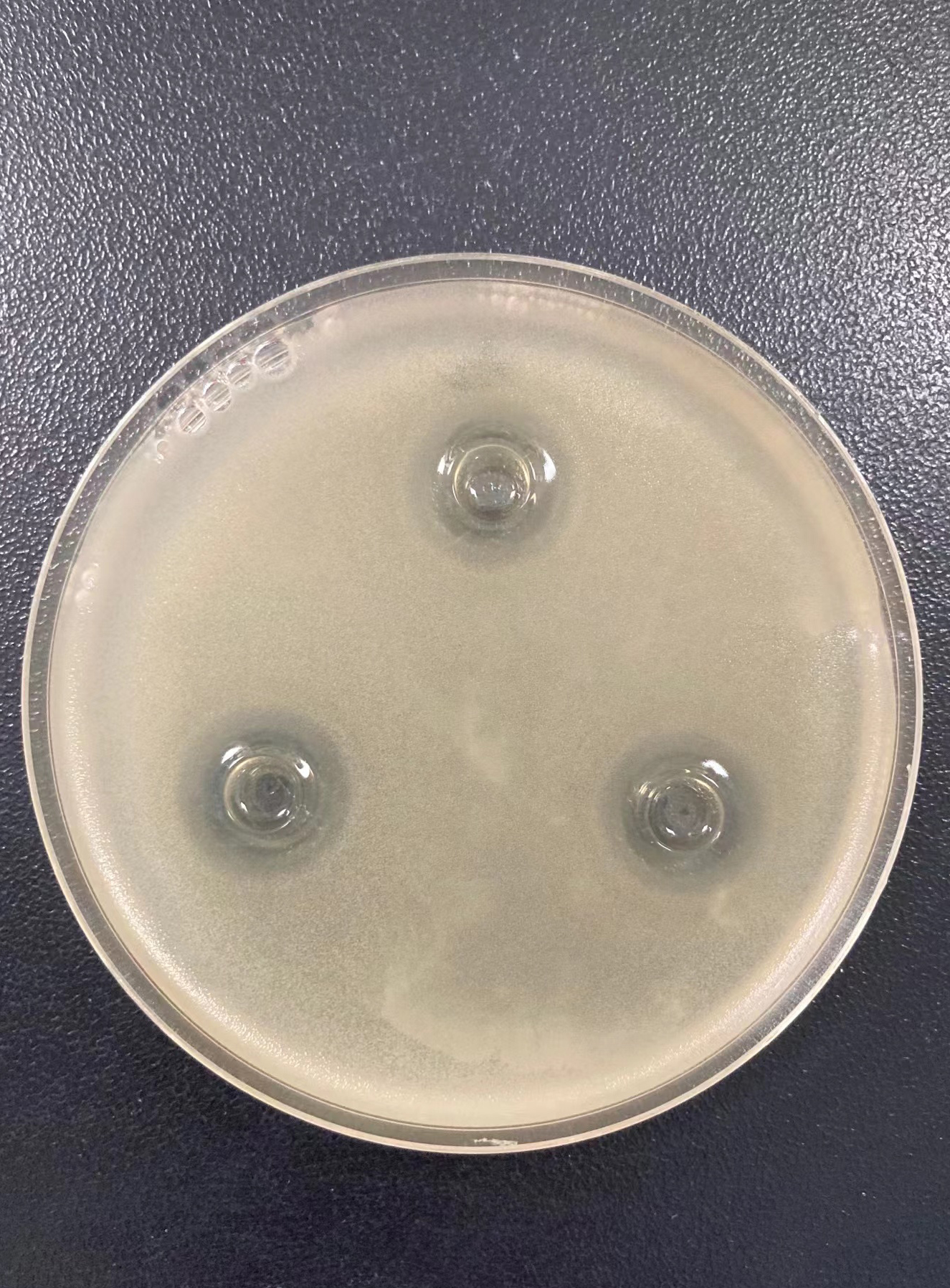

Supplement: Figure 2—figure supplement 1—source data 2. [file elife-93423-fig2-figsupp1-data2.zip › Figure 2—figure supplement 1—source data 2/Figure 2—figure supplement 1—source data 2/P. multocida PM002.jpg]

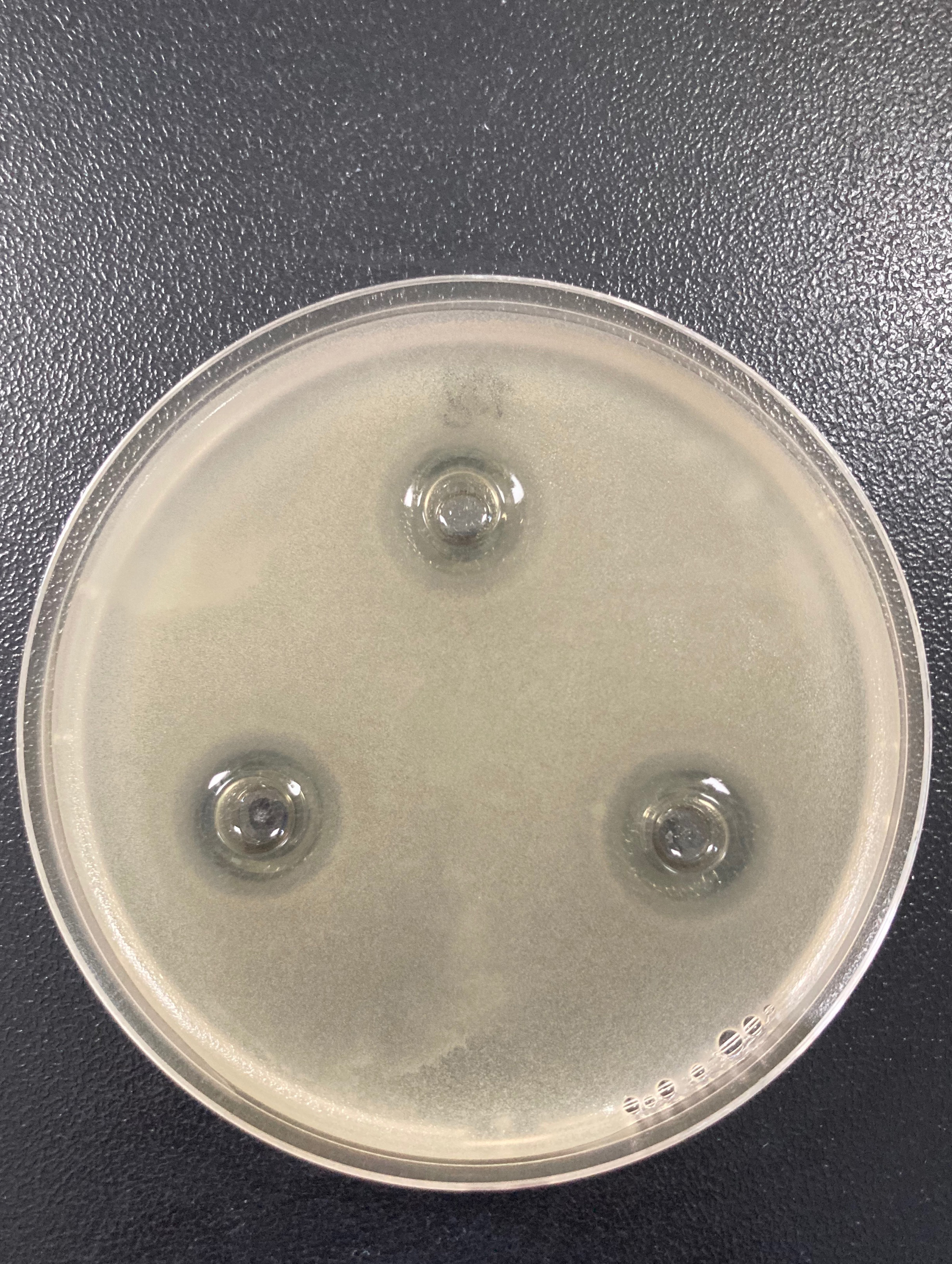

Supplement: Figure 2—figure supplement 1—source data 2. [file elife-93423-fig2-figsupp1-data2.zip › Figure 2—figure supplement 1—source data 2/Figure 2—figure supplement 1—source data 2/P. multocida PM008.jpg]

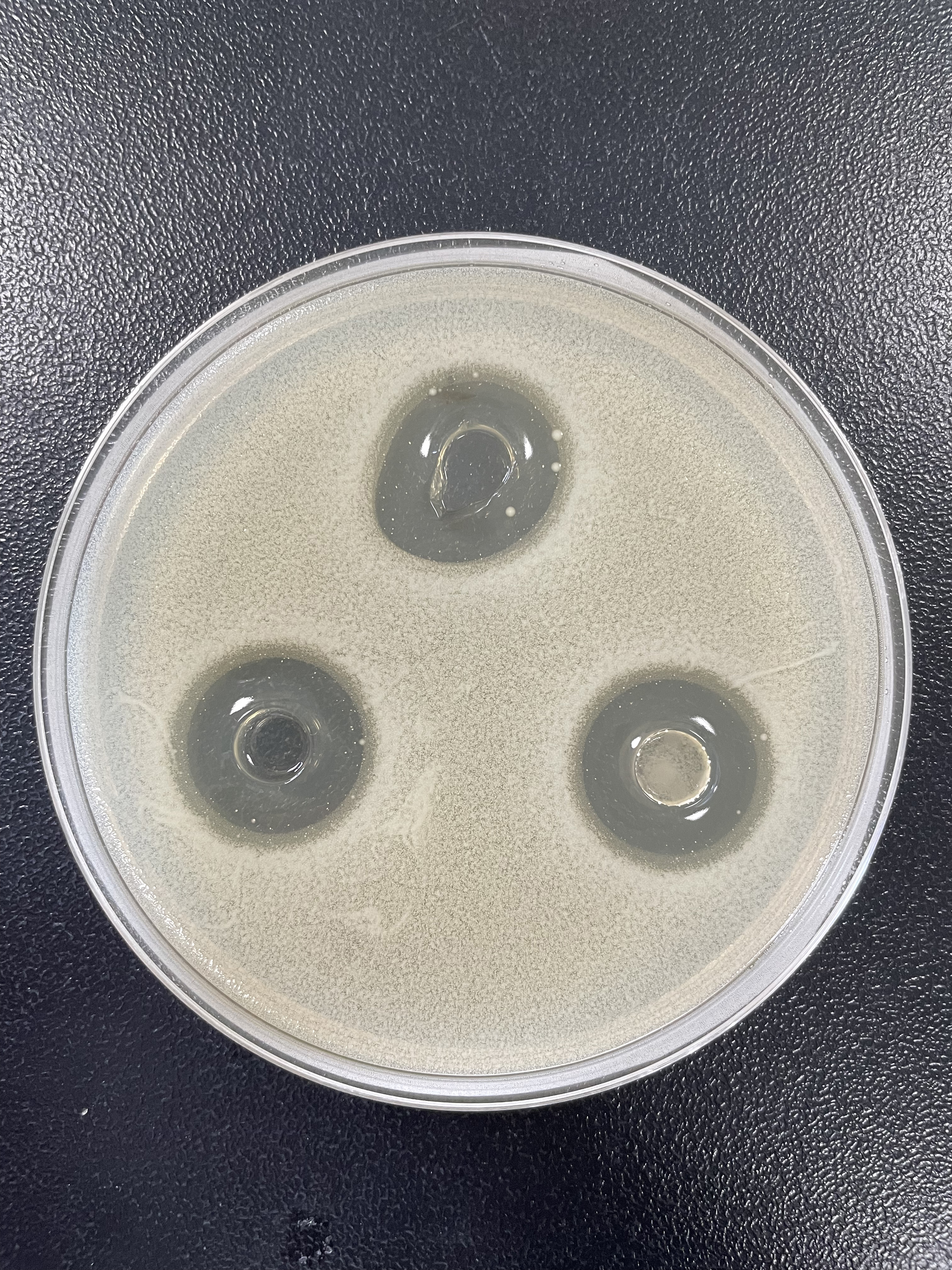

Supplement: Figure 2—figure supplement 1—source data 2. [file elife-93423-fig2-figsupp1-data2.zip › Figure 2—figure supplement 1—source data 2/Figure 2—figure supplement 1—source data 2/S. aureus ATCC29213.jpg]

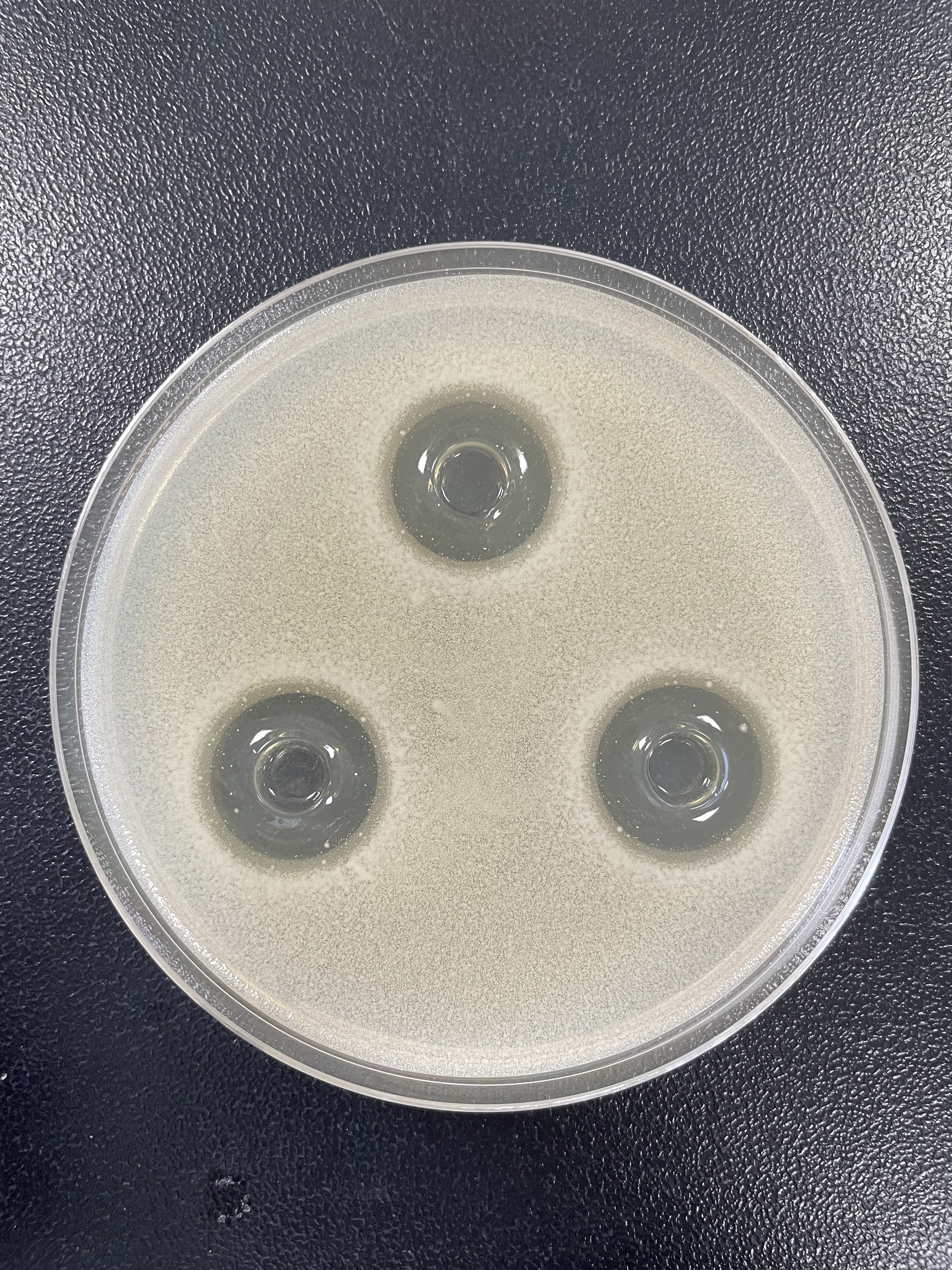

Supplement: Figure 2—figure supplement 1—source data 2. [file elife-93423-fig2-figsupp1-data2.zip › Figure 2—figure supplement 1—source data 2/Figure 2—figure supplement 1—source data 2/S. aureus ATCC43300.jpg]

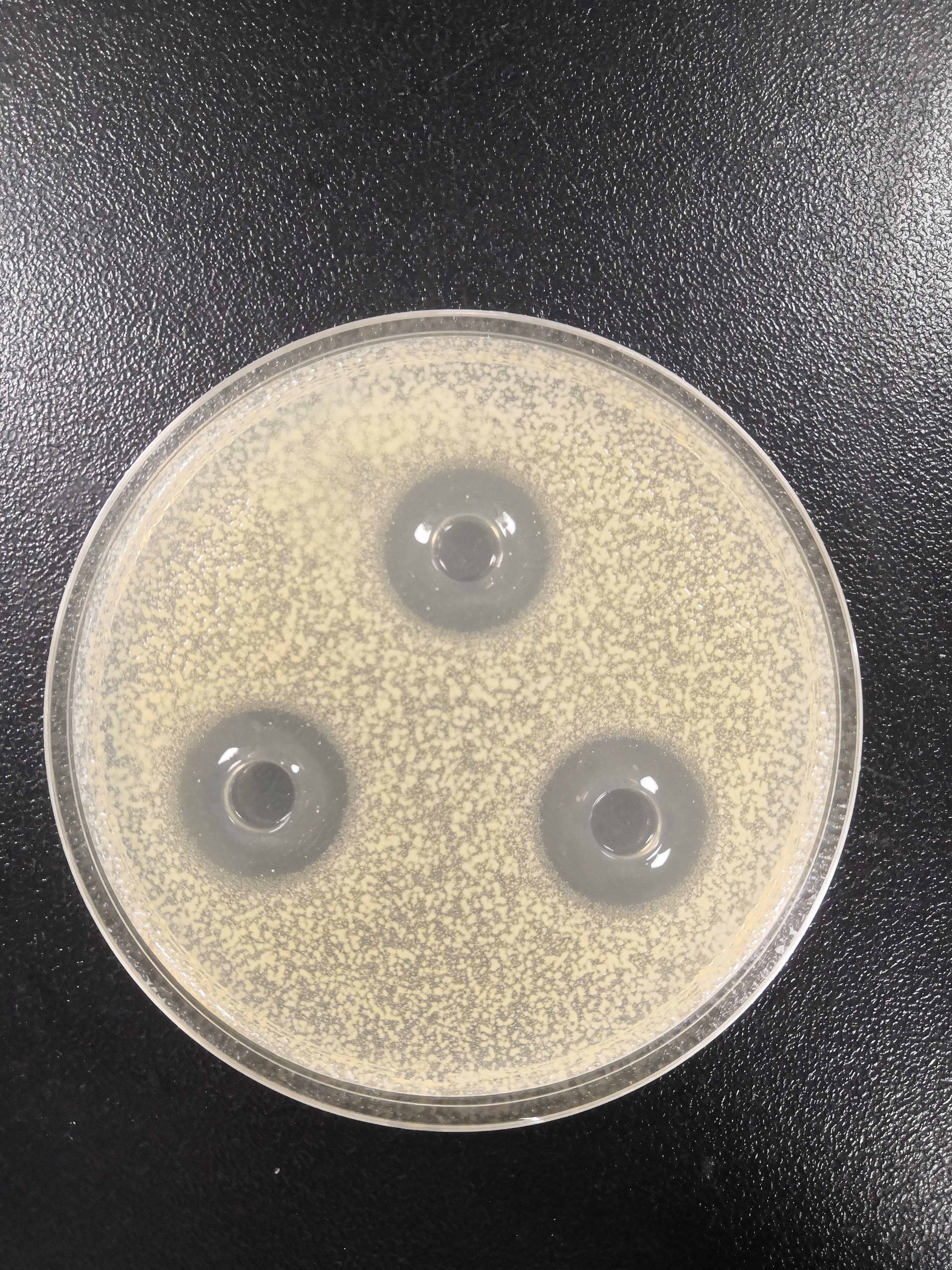

Supplement: Figure 2—figure supplement 1—source data 2. [file elife-93423-fig2-figsupp1-data2.zip › Figure 2—figure supplement 1—source data 2/Figure 2—figure supplement 1—source data 2/S. aureus S21.jpg]

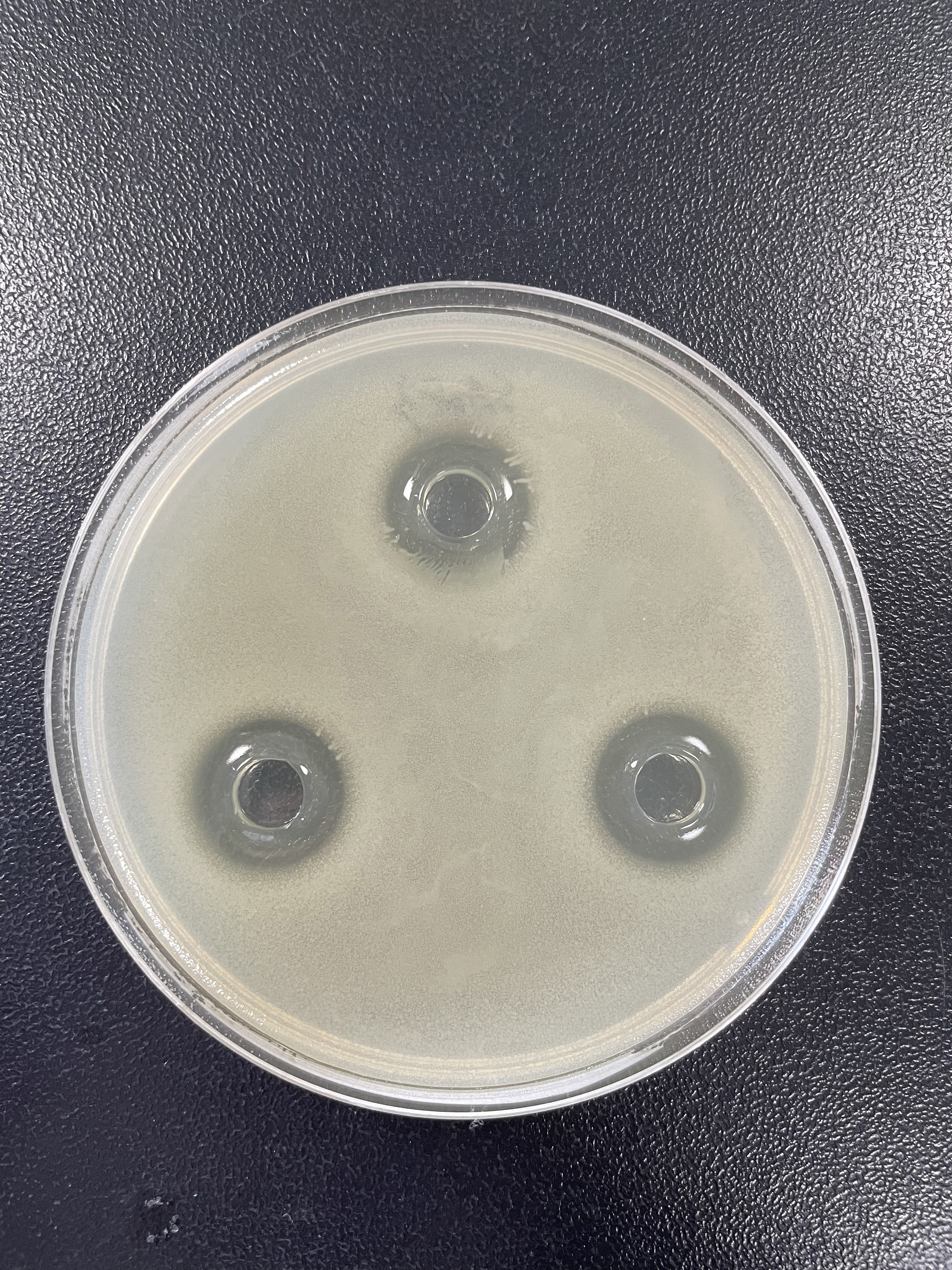

Supplement: Figure 2—figure supplement 1—source data 2. [file elife-93423-fig2-figsupp1-data2.zip › Figure 2—figure supplement 1—source data 2/Figure 2—figure supplement 1—source data 2/S. Enteritidis SE006.jpg]

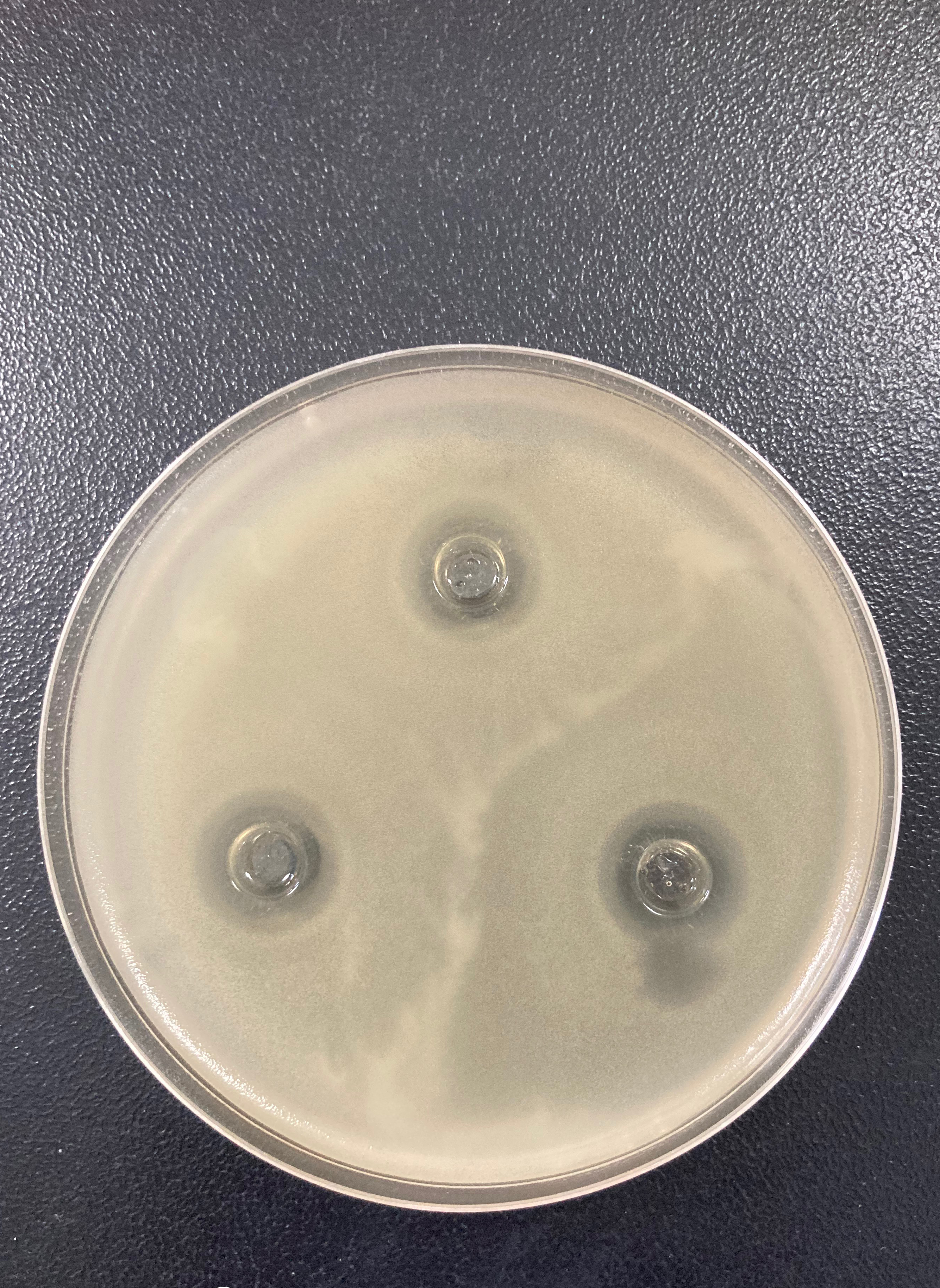

Supplement: Figure 2—figure supplement 1—source data 2. [file elife-93423-fig2-figsupp1-data2.zip › Figure 2—figure supplement 1—source data 2/Figure 2—figure supplement 1—source data 2/S. suis SC19.jpg]

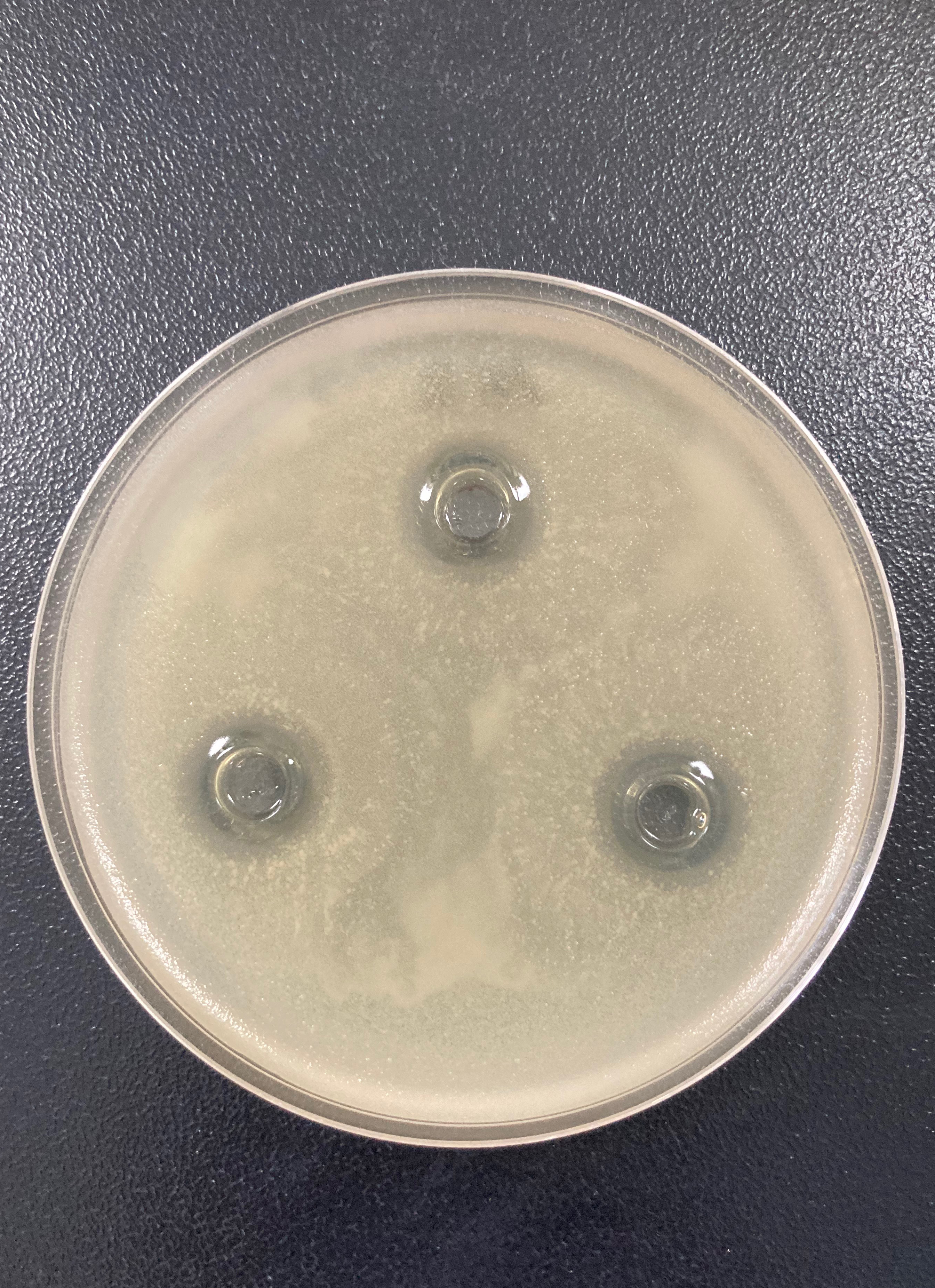

Supplement: Figure 2—figure supplement 1—source data 2. [file elife-93423-fig2-figsupp1-data2.zip › Figure 2—figure supplement 1—source data 2/Figure 2—figure supplement 1—source data 2/S. suis SS006.jpg]

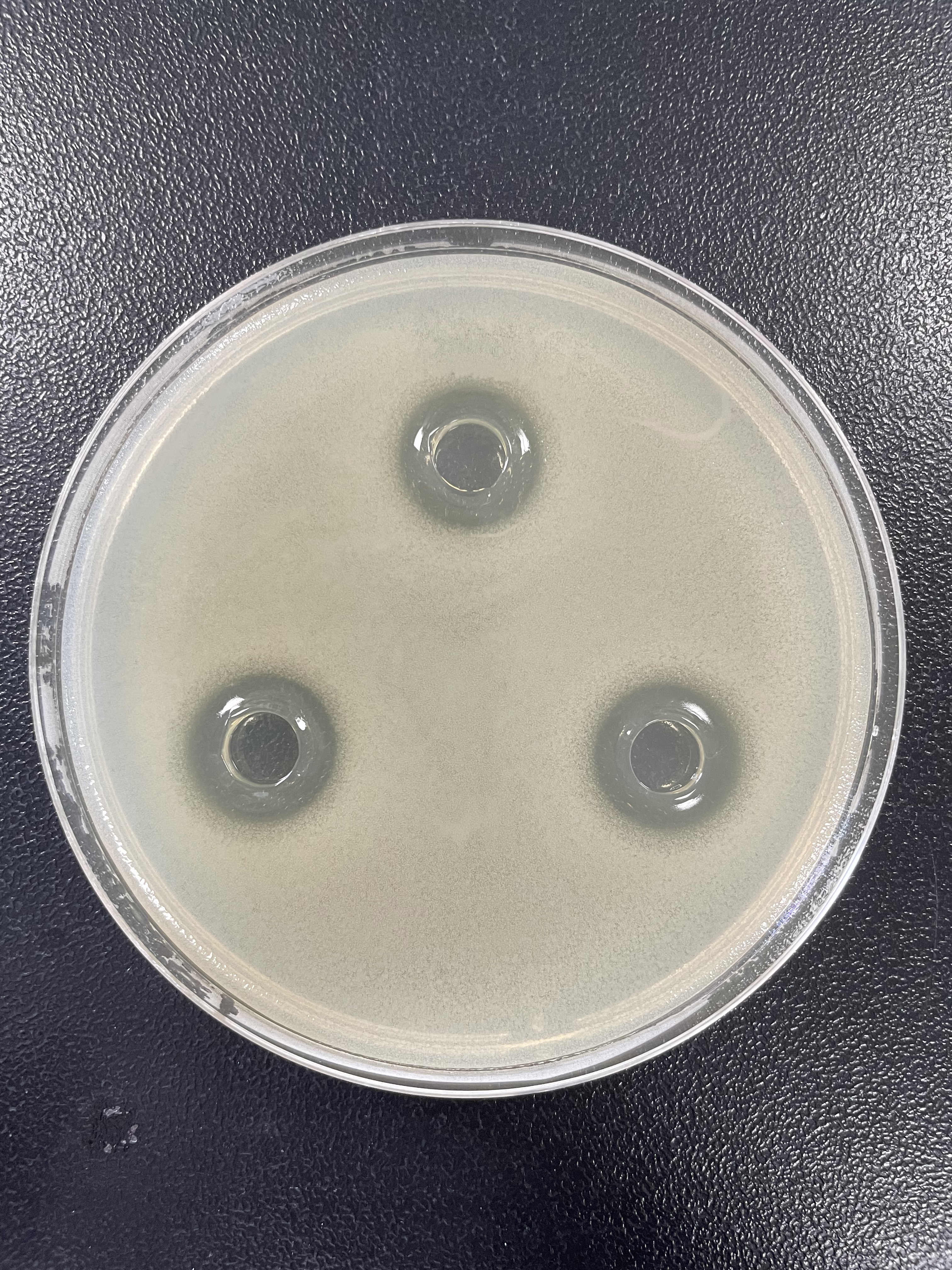

Supplement: Figure 2—figure supplement 1—source data 2. [file elife-93423-fig2-figsupp1-data2.zip › Figure 2—figure supplement 1—source data 2/Figure 2—figure supplement 1—source data 2/S. Typhimurium ATCC14028.jpg]

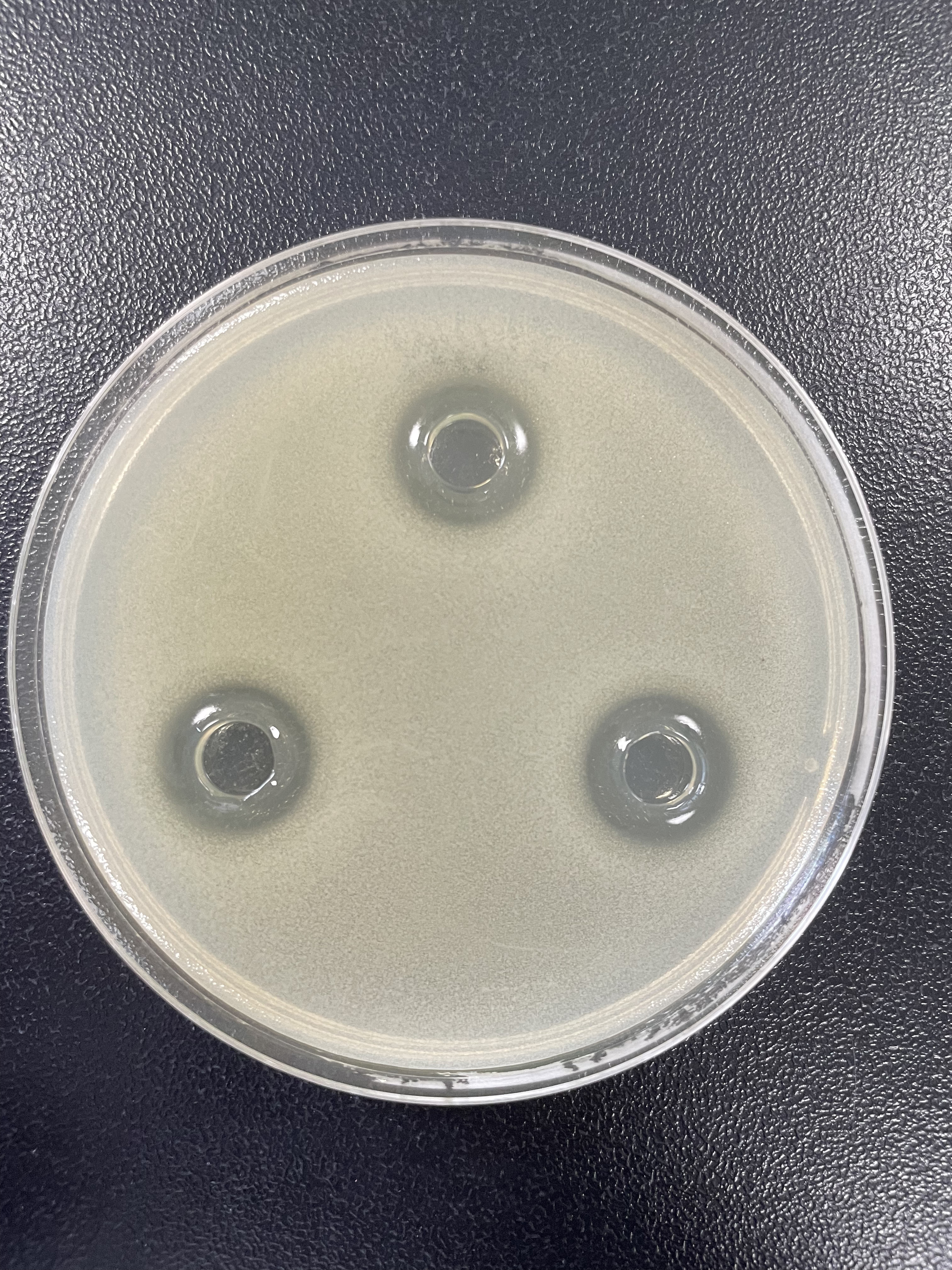

Supplement: Figure 2—figure supplement 1—source data 2. [file elife-93423-fig2-figsupp1-data2.zip › Figure 2—figure supplement 1—source data 2/Figure 2—figure supplement 1—source data 2/S. Typhimurium SL1344.jpg]

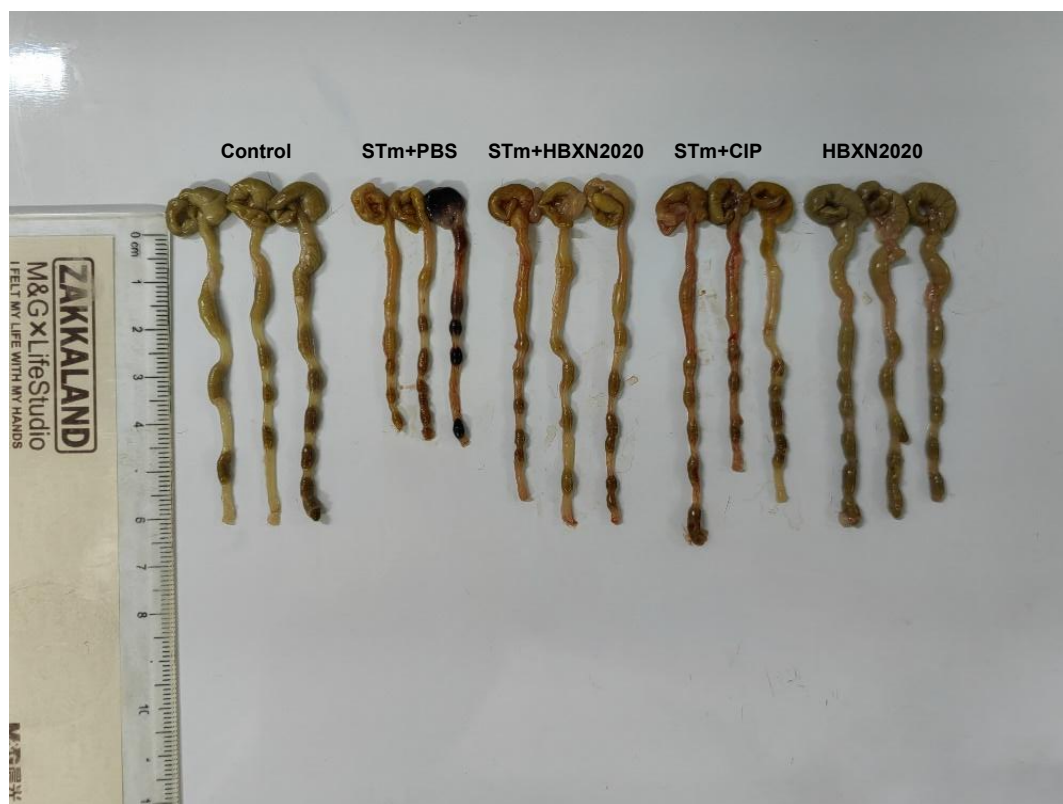

Fig. 5H. Colonic tissue images of mice in Control, STm+PBS, STm+HBXN2020, STm+CIP and HBXN2020 groups.

Supplement: Figure 5—source data 2. [file elife-93423-fig5-data2.zip › Figure 5—source data 2/Figure 5—source data 2.pdf]

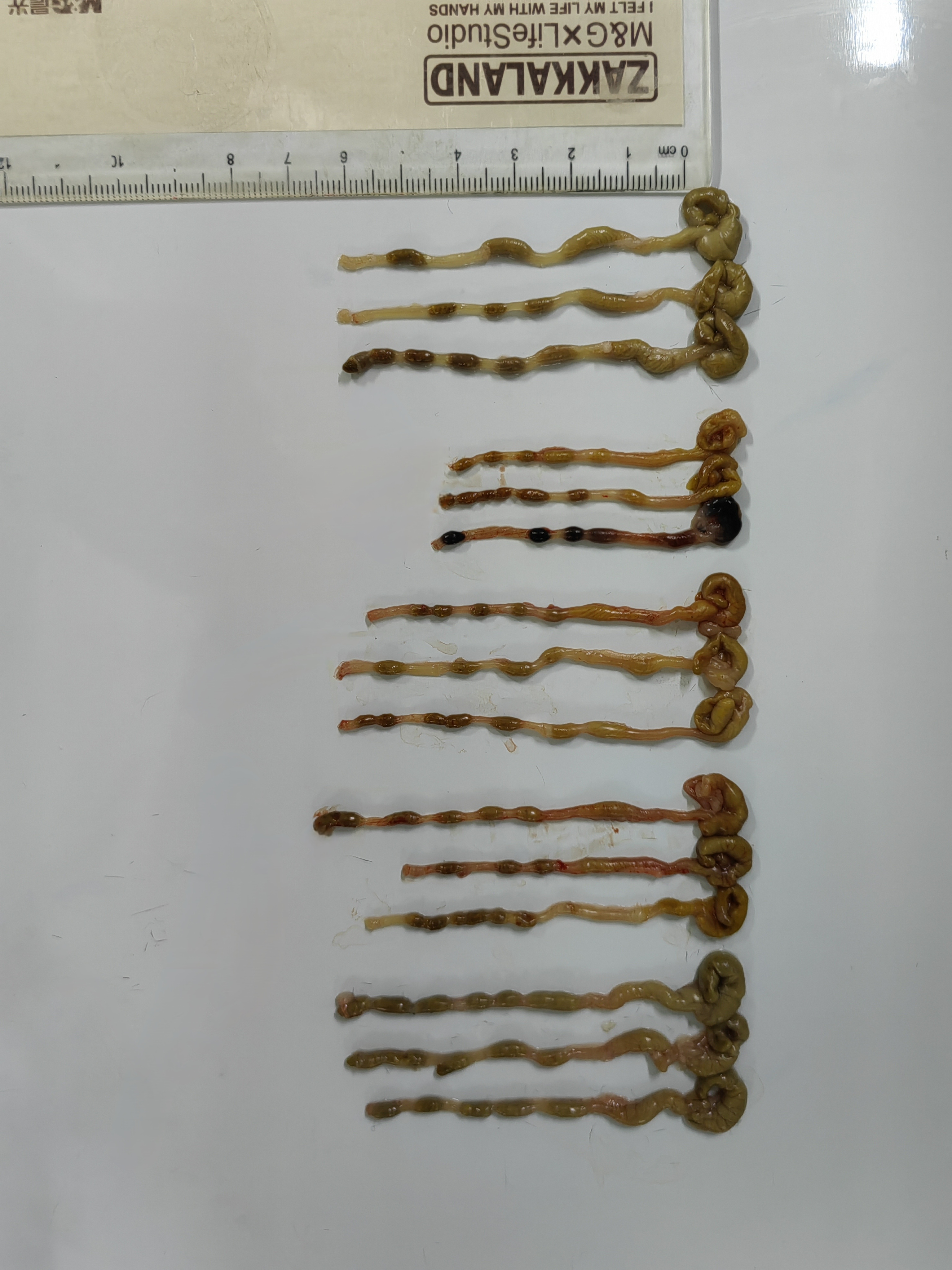

Supplement: Figure 5—source data 3. [file elife-93423-fig5-data3.zip › Figure 5—source data 3/Figure 5—source data 3/Fig. 5H.jpg]

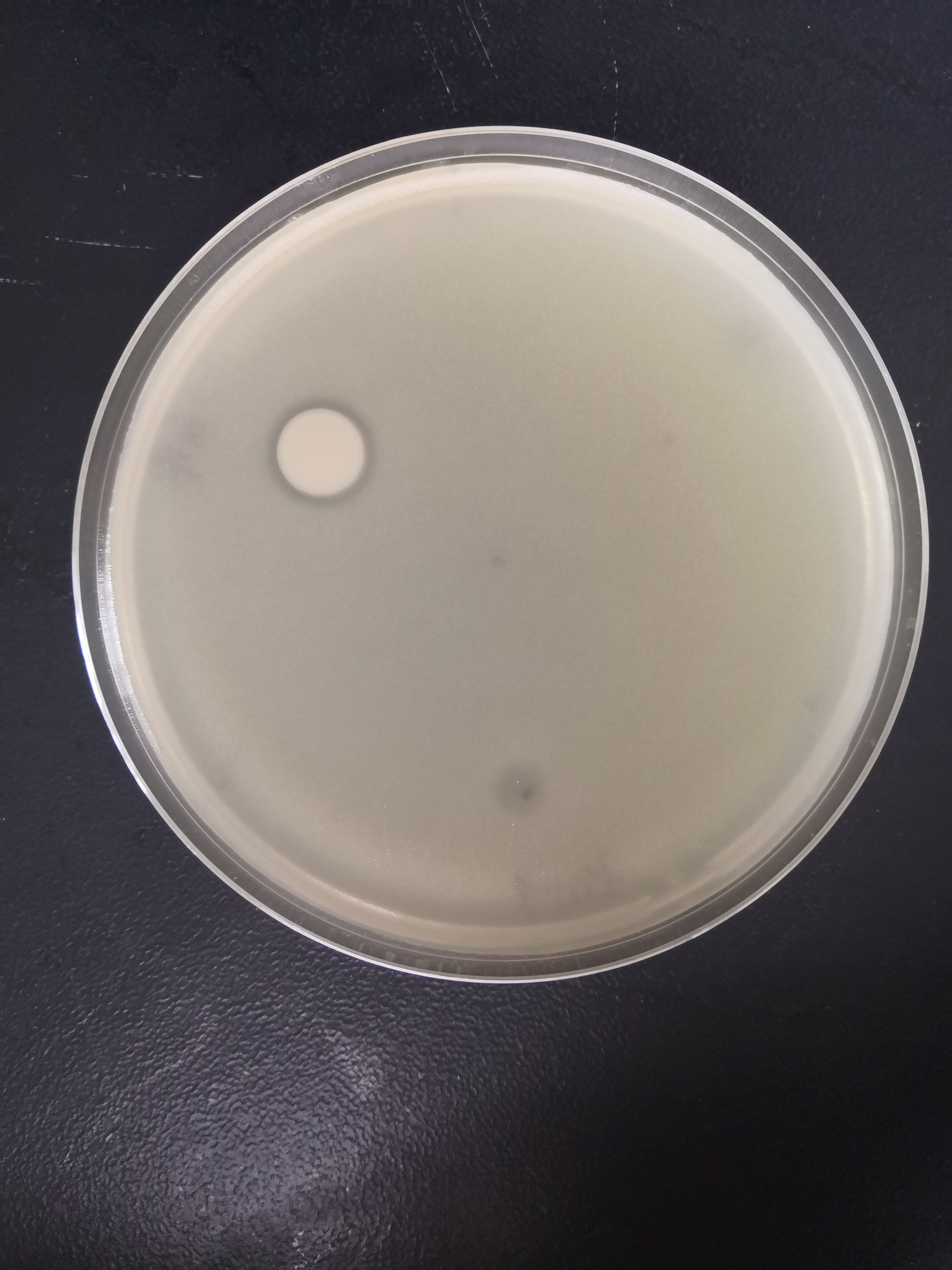

Supplement: Figure 5—figure supplement 1—source data 3. [file elife-93423-fig5-figsupp1-data3.zip › Figure 5—figure supplement 1—source data 3/Figure 5—figure supplement 1—source data 3/A.jpg]

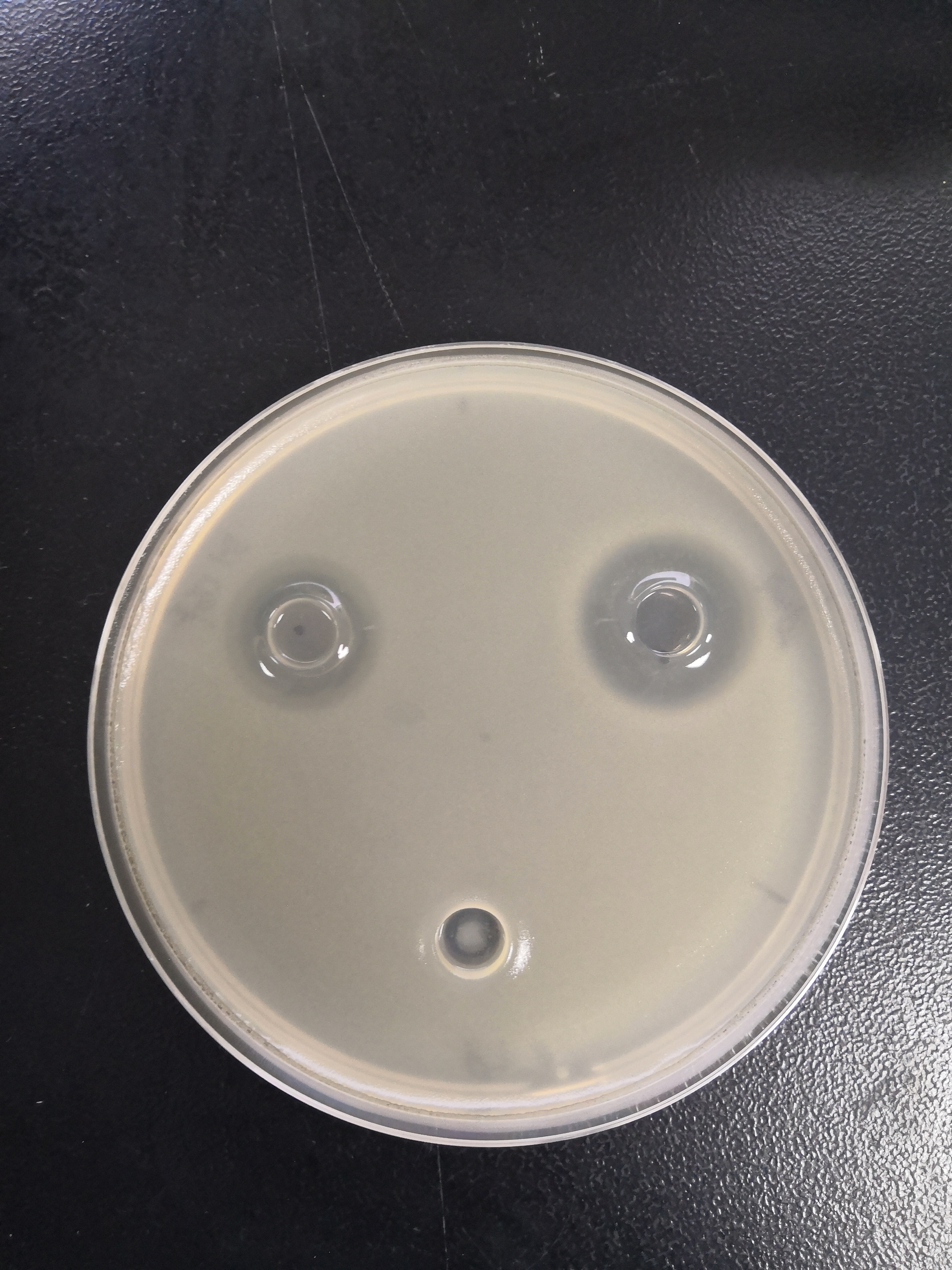

Supplement: Figure 5—figure supplement 1—source data 3. [file elife-93423-fig5-figsupp1-data3.zip › Figure 5—figure supplement 1—source data 3/Figure 5—figure supplement 1—source data 3/B.jpg]

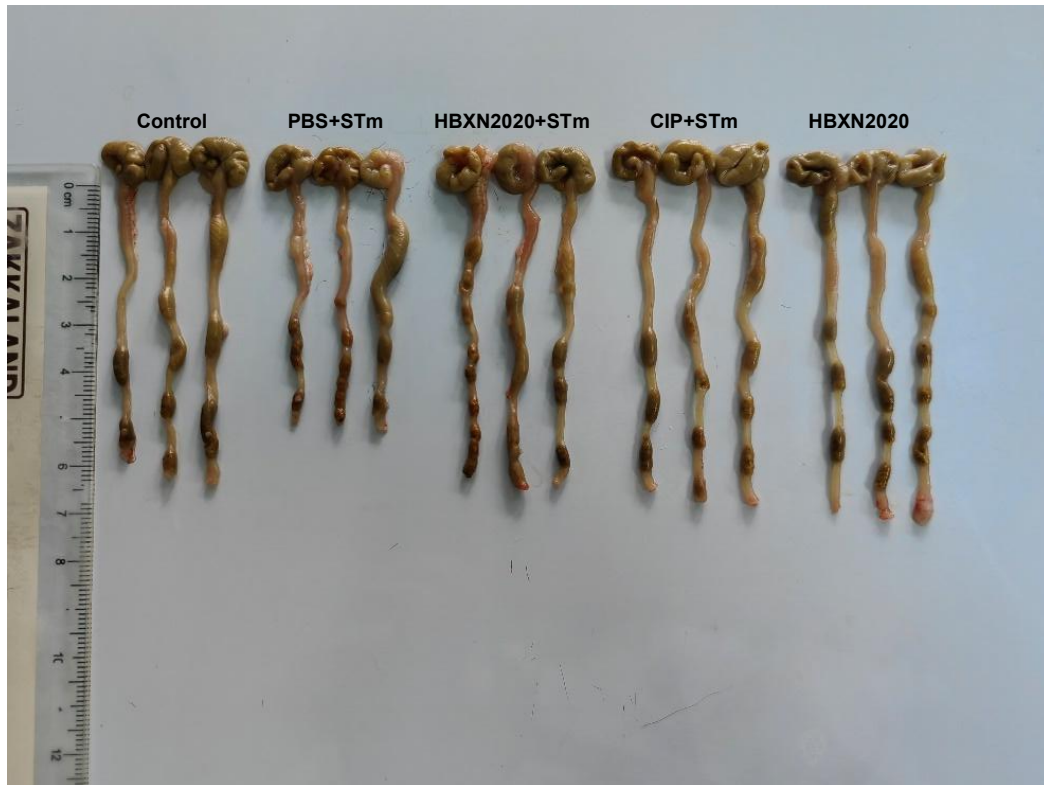

Fig. 8D. Colonic tissue images of mice in Control, PBS+STm, HBXN2020+STm, CIP+STm and HBXN2020 groups.

Supplement: Figure 8—source data 2. [file elife-93423-fig8-data2.zip › Figure 8—source data 2/Figure 8—source data 2.pdf]

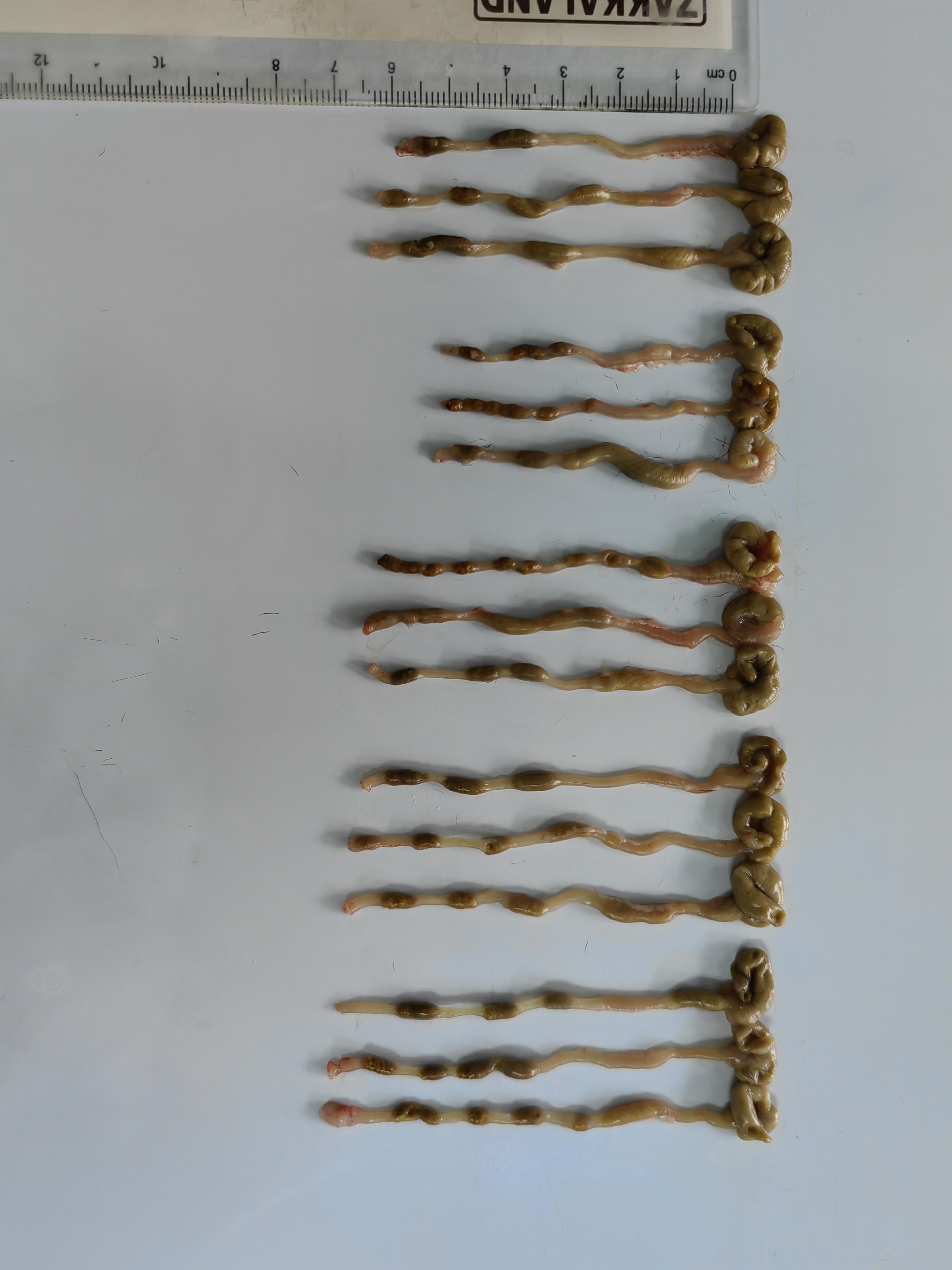

Supplement: Figure 8—source data 3. [file elife-93423-fig8-data3.zip › Figure 8—source data 3/Figure 8—source data 3/Fig. 8D.jpg]
