# Supplementary material for: Bacillus velezensis HBXN2020 alleviates Salmonella Typhimurium infection in mice by improving intestinal barrier integrity and reducing inflammation
Source: eLife. 2024 Nov 19;13:RP93423. doi: 10.7554/eLife.93423 (PMC11575897; doi:10.7554/eLife.93423)
Supplement: Figure 2—figure supplement 1—source data 1. [file elife-93423-fig2-figsupp1-data1.zip › Figure 2—figure supplement 1—source data 1/Figure 2—figure supplement 1—source data 1.pdf]

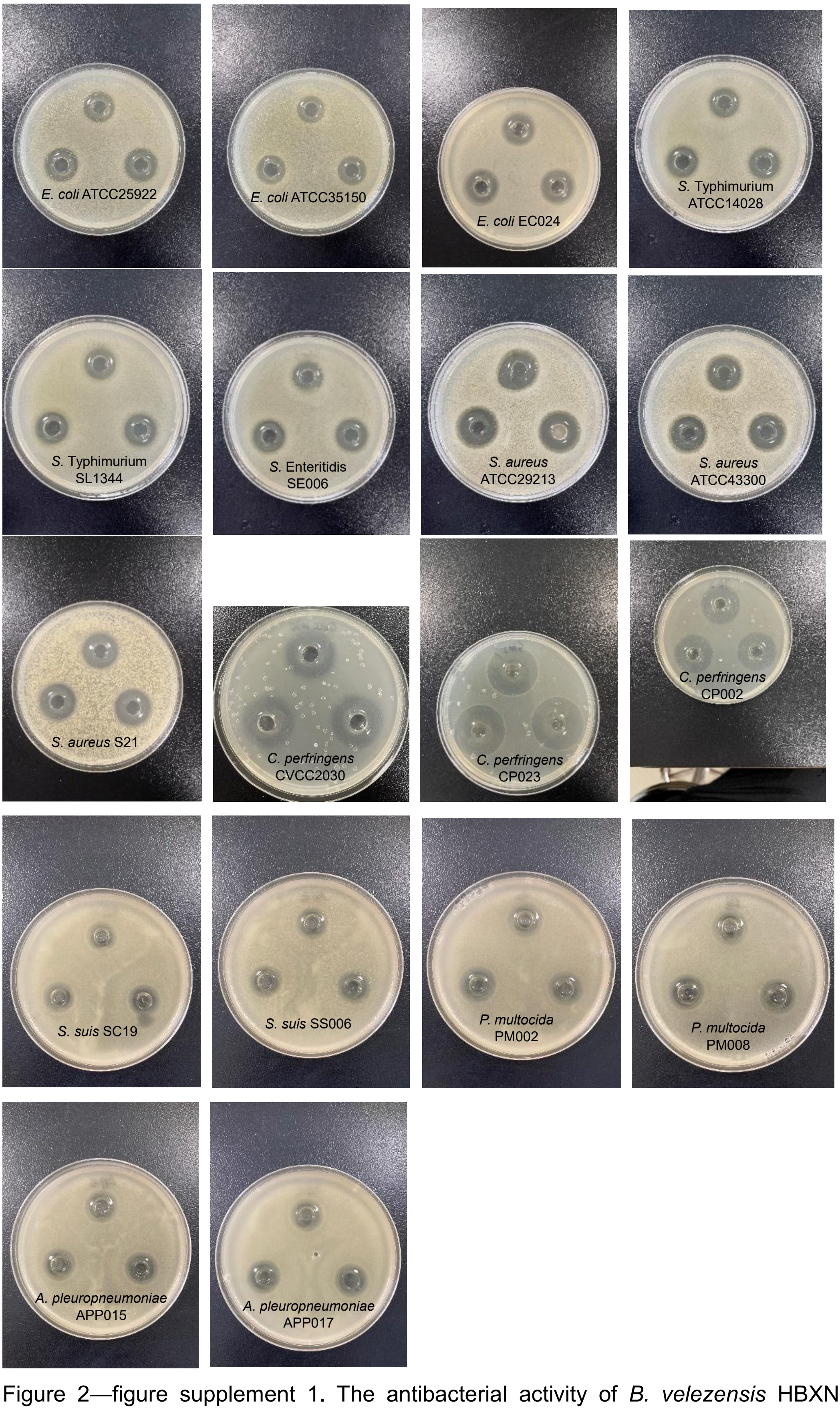

Figure 2—figure supplement 1. The antibacterial activity of *B. velezensis* HBXN2020 fermentation supernatant *in vitro*.
