# Supplementary material for: Bacillus velezensis HBXN2020 alleviates Salmonella Typhimurium infection in mice by improving intestinal barrier integrity and reducing inflammation
Source: eLife. 2024 Nov 19;13:RP93423. doi: 10.7554/eLife.93423 (PMC11575897; doi:10.7554/eLife.93423)
Supplement: Figure 5—figure supplement 1—source data 2. [file elife-93423-fig5-figsupp1-data2.zip › Figure 5—figure supplement 1—source data 2/Figure 5—figure supplement 1—source data 2.pdf]

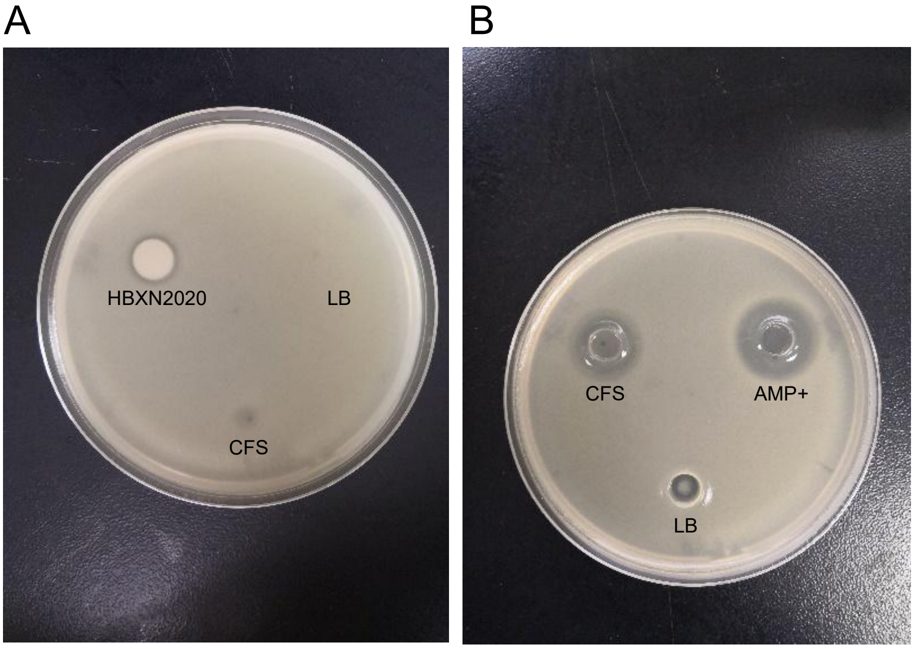

Figure 5—figure supplement 1A, B. The antibacterial activity of *B. velezensis* HBXN2020 and its fermentation supernatant against *S*. Typhimurium ATCC14028 in solid culture media.
