# Supplementary material for: Bacillus velezensis HBXN2020 alleviates Salmonella Typhimurium infection in mice by improving intestinal barrier integrity and reducing inflammation
Source: eLife. 2024 Nov 19;13:RP93423. doi: 10.7554/eLife.93423 (PMC11575897; doi:10.7554/eLife.93423)
Supplement: Supplementary file 1. [file elife-93423-supp1.docx]

**Supplementary** **file 1. Determination of antibacterial activity of different *Bacillus***

| **Bacteria**  **and serovars** | **Strain no.** | 1. ***subtilis***   **BSH1** | ***B. velezensis***  **HBXN2020** | ***B. amyloliquefaciens***  **6-1** | 1. ***licheniformis***   **BSK14** |
| --- | --- | --- | --- | --- | --- |
| *E. coli* | EC006 | - | ++ | + | - |
|  | EC016 | - | +++ | + | - |
|  | EC022 | - | ++ | - | - |
|  | EC024 | - | +++ | ++ | - |
|  | ATCC  35150 | - | +++ | ++ | - |
|  | ATCC  25922 | - | +++ | ++ | - |
| *S.* Typhimurium | ST001 | - | +++ | ++ | - |
|  | ST002 | - | ++ | +++ | - |
|  | ST003 | - | + | + | - |
|  | ST004 | - | +++ | + | - |
|  | ST005 | - | +++ | ++ | - |
|  | ST006 | - | +++ | +++ | - |
|  | ST007 | - | +++ | ++ | - |
|  | SL1344 | - | +++ | ++ | - |
|  | ATCC  14028 | - | +++ | + | - |
| 1. Enteritidis | SE001 | - | +++ | ++ | - |
|  | SE002 | - | +++ | ++ | - |
|  | SE003 | - | +++ | ++ | - |
|  | SE004 | - | +++ | +++ | - |
|  | SE005 | - | ++ | ++ | - |
|  | SE006 | - | +++ | ++ | - |
| *S. aureus* | S1 | ++ | +++ | +++ | +++ |
|  | S2 | ++ | +++ | +++ | +++ |
|  | S3 | ++ | +++ | +++ | ++ |
|  | S4 | ++ | +++ | +++ | ++ |
|  | S5 | ++ | +++ | +++ | +++ |
|  | S6 | +++ | +++ | +++ | +++ |
|  | S10 | ++ | ++ | +++ | +++ |
|  | S11 | ++ | +++ | +++ | ++ |
|  | S12 | ++ | ++ | +++ | ++ |
|  | S13 | ++ | ++ | + | +++ |
|  | S14 | ++ | ++ | ++ | +++ |
|  | S15 | +++ | +++ | +++ | ++ |
|  | S16 | +++ | ++ | ++ | ++ |
|  | S17 | +++ | ++ | +++ | ++ |
|  | S18 | ++ | +++ | +++ | +++ |
|  | S19 | ++ | ++++ | +++ | +++ |
|  | S20 | ++ | ++ | +++ | ++ |
|  | S21 | ++ | +++ | ++ | +++ |
|  | ATCC  43300 | ++ | +++ | +++ | +++ |
|  | ATCC  25923 | ++ | +++ | +++ | ++ |
|  | ATCC  29213 | +++ | +++ | +++ | ++ |
| *S. suis* | SS006 | ++ | +++ | +++ | + |
|  | SS12 | ++ | +++ | +++ | ++ |
|  | SS54 | +++ | ++ | ++ | ++ |
|  | SS55 | ++ | ++ | ++ | ++ |
|  | SS57 | ++ | ++ | ++ | ++ |
|  | SS58 | +++ | +++ | +++ | ++ |
|  | SS59 | ++ | +++ | ++ | ++ |
|  | SS60 | +++ | ++ | +++ | +++ |
|  | SS61 | ++ | +++ | +++ | ++ |
|  | SS62 | ++ | ++ | ++ | ++ |
|  | SS63 | ++ | ++ | ++ | +++ |
|  | SS64 | ++ | ++ | ++ | ++ |
|  | SC19 | ++ | ++ | +++ | ++ |
| *C. perfringens* | CP001 | ++ | +++ | +++ | +++ |
|  | CP002 | +++ | ++++ | +++ | +++ |
|  | CP003 | ++ | +++ | +++ | +++ |
|  | CP004 | ++ | +++ | +++ | +++ |
|  | CP005 | ++ | +++ | +++ | +++ |
|  | CP006 | +++ | +++ | +++ | +++ |
|  | CP007 | ++ | +++ | +++ | +++ |
|  | CP008 | ++ | +++ | +++ | +++ |
|  | CP009 | ++ | +++ | +++ | +++ |
|  | CP010 | ++ | +++ | +++ | +++ |
|  | CP011 | ++ | +++ | +++ | +++ |
|  | CP012 | ++ | +++ | +++ | +++ |
|  | CP013 | ++ | +++ | +++ | +++ |
|  | CP014 | ++ | +++ | +++ | +++ |
|  | CP015 | ++ | +++ | +++ | +++ |
|  | CP016 | ++ | +++ | +++ | +++ |
|  | CP017 | ++ | +++ | +++ | +++ |
|  | CP018 | ++ | +++ | +++ | +++ |
|  | CP023 | ++ | ++++ | +++ | +++ |
|  | CVCC  2030 | ++ | ++++ | +++ | ++++ |
| *A. pleuropneumoniae* | APP015 | - | ++ | + | - |
|  | APP016 | - | ++ | ++ | - |
|  | APP017 | - | ++ | ++ | - |
|  | APP018 | - | ++ | + | - |
| *P. multocida* | PM002 | - | +++ | ++ | - |
|  | PM008 | - | +++ | +++ | - |

Note: -, No antibacterial activity; +, 0 < bacteriostatic diameter ≤ 5; ++, 5 < bacteriostatic diameter ≤ 15; +++, 15 < bacteriostatic diameter ≤ 20；++++, 20 < bacteriostatic diameter.
