# Supplementary material for: Bacillus velezensis HBXN2020 alleviates Salmonella Typhimurium infection in mice by improving intestinal barrier integrity and reducing inflammation
Source: eLife. 2024 Nov 19;13:RP93423. doi: 10.7554/eLife.93423 (PMC11575897; doi:10.7554/eLife.93423)
Supplement: Supplementary file 2. — (a) HBXN2020 genome features. (b) Clusters of secondary metabolic synthesis genes in HBXN2020. [file elife-93423-supp2.docx]

**Supplementary file 2. HBXN2020 genome features and clusters of secondary metabolic synthesis genes.**

**Supplementary file 2a. HBXN2020 genome features**

| Features | Value |
| --- | --- |
| Genome size (bp) | 3,929,792 |
| G+C content% | 46.5 |
| Protein coding genes | 3,744 |
| Gene average length (bp) | 928.06 |
| Plasmid | 0 |
| rRNA | 27 |
| tRNA | 86 |

**Supplementary file 2b. Clusters of secondary metabolic synthesis genes in HBXN2020**

| BGC | BGC type | Length (bp) | BGC content (% Similarity) |
| --- | --- | --- | --- |
| 1 | NAD(P)/FAD-dependent oxidoreductase | 63978 | Surfactin (82%) |
| 2 | PKS-like | 41254 | Butirosin A / Butirosin B (7%) |
| 3 | Hypothetical protein | 17409 | Unknown |
| 4 | Lanthipeptide | 28889 | Unknown |
| 5 | 1-phosphofructokinase | 87836 | Macrolactin H (100%) |
| 6 | Competence/damage-inducible protein A | 109575 | Bacillaene (100%) |
| 7 | Zinc-binding alcohol dehydrogenase family protein | 134311 | Fengycin (100%) |
| 8 | LysM peptidoglycan-binding domain-containing protein | 19553 | Unknown |
| 9 | Terpene | 21884 | Unknown |
| 10 | T3PKS | 41101 | Unknown |
| 11 | TransAT-PKS-like | 106183 | Difficidin (100%) |
| 12 | NRPS | 51792 | Bacillibactin (100%) |
| 13 | Other | 41419 | Bacilysin (100%) |
