# Supplementary material for: Bacillus velezensis HBXN2020 alleviates Salmonella Typhimurium infection in mice by improving intestinal barrier integrity and reducing inflammation
Source: eLife. 2024 Nov 19;13:RP93423. doi: 10.7554/eLife.93423 (PMC11575897; doi:10.7554/eLife.93423)
Supplement: Supplementary file 3. — (a) The bacterial strains used in this study. (b) The primer sequences used for the RT-qPCR. (c) Disease activity index (DAI) parameters and their associated scoring schemes. [file elife-93423-supp3.docx]

**Supplementary file 3. The bacterial strains, RT-qPCR primers and disease activity index scoring schemes in this study.**

**Supplementary file 3a. The bacterial strains used in this study**

| **Strains** | **Strain ID number** | **Source** |
| --- | --- | --- |
| *Bacillus velezensis* (*B. velezensis*, NCBI no. CP119399) | HBXN2020 | Lab stock |
| *Escherischia coli* (*E. coli*) | ATCC25922 | ATCC |
|  | ATCC35150 | ATCC |
|  | EC024 | Lab stock |
|  | EC022 | Lab stock |
|  | EC016 | Lab stock |
|  | EC006 | Lab stock |
| *Salmonella enterica* serovar Typhimurium (*S.* Typhimurium, carry pET28a (+), kanamycin resistance) | ATCC14028 | ATCC |
|  | SL1344 | Lab stock |
|  | ST001 | Lab stock |
|  | ST002 | Lab stock |
|  | ST003 | Lab stock |
|  | ST004 | Lab stock |
|  | ST005 | Lab stock |
|  | ST006 | Lab stock |
|  | ST007 | Lab stock |
| *Salmonella enterica* serovar Enteritidis (*S.* Enteritidis) | SE006 | Lab stock |
|  | SE001 | Lab stock |
|  | SE002 | Lab stock |
|  | SE003 | Lab stock |
|  | SE004 | Lab stock |
|  | SE005 | Lab stock |
| *Staphylococcus aureus* (*S. aureus*) | ATCC29213 | ATCC |
|  | ATCC43300 | ATCC |
|  | ATCC25923 | ATCC |
|  | S21 | Lab stock |
|  | S1 | Lab stock |
|  | S2 | Lab stock |
|  | S3 | Lab stock |
|  | S4 | Lab stock |
|  | S5 | Lab stock |
|  | S6 | Lab stock |
|  | S10 | Lab stock |
|  | S11 | Lab stock |
|  | S12 | Lab stock |
|  | S13 | Lab stock |
|  | S14 | Lab stock |
|  | S15 | Lab stock |
|  | S16 | Lab stock |
|  | S17 | Lab stock |
|  | S18 | Lab stock |
|  | S19 | Lab stock |
|  | S20 | Lab stock |
| *Clostridium perfringens* (*C. perfringens*) | CVCC2030 | Lab stock |
|  | CP023 | Lab stock |
|  | CP001 | Lab stock |
|  | CP002 | Lab stock |
|  | CP003 | Lab stock |
|  | CP004 | Lab stock |
|  | CP005 | Lab stock |
|  | CP006 | Lab stock |
|  | CP007 | Lab stock |
|  | CP008 | Lab stock |
|  | CP009 | Lab stock |
|  | CP010 | Lab stock |
|  | CP011 | Lab stock |
|  | CP012 | Lab stock |
|  | CP013 | Lab stock |
|  | CP014 | Lab stock |
|  | CP015 | Lab stock |
|  | CP016 | Lab stock |
|  | CP017 | Lab stock |
|  | CP018 | Lab stock |
| *Streptococcus suis* (*S. suis*) | SC19 | Lab stock |
|  | SS006 | Lab stock |
|  | SS12 | Lab stock |
|  | SS54 | Lab stock |
|  | SS55 | Lab stock |
|  | SS57 | Lab stock |
|  | SS58 | Lab stock |
|  | SS59 | Lab stock |
|  | SS60 | Lab stock |
|  | SS61 | Lab stock |
|  | SS62 | Lab stock |
|  | SS63 | Lab stock |
|  | SS64 | Lab stock |
| *Pasteurella multocida* (*P. multocida*) | PM002 | Lab stock |
|  | PM008 | Lab stock |
| *Actinobacillus pleuropneumoniae* (*A. pleuropneumoniae*) | APP015 | Lab stock |
|  | APP016 | Lab stock |
|  | APP017 | Lab stock |
|  | APP018 | Lab stock |

ATCC, American Type Culture Collection

**Supplementary file 3b. The primer sequences used for the RT-qPCR**

| Genes | Primer sequence (5’ to 3’) |
| --- | --- |
| *16S rDNA* | Forward: AGAGTTTGATCCTGGCTCAG |
|  | Reverse: GGTTACCTTGTTACGACTT |
| *gyrB* | Forward: ATGGCTATGGAACAGCAGCAAAATAG |
|  | Forward:AATATCAAGATTTTTCACGTATCTGGCGT |
| *Tnfa* | Forward: CCACGCTCTTCTGTCTACTG |
|  | Reverse: ACTTGGTGGTTTGCTACGA |
| *Il1b* | Forward: ACCTGTGTCTTTCCCGTGG |
|  | Reverse: TCATCTCGGAGCCTGTAGTG |
| *Il6* | Forward: GAGCCCACCAAGAACGATA |
|  | Reverse: TTGTCACCAGCATCAGTCC |
| *Il10* | Forward: TGGACAACATACTGCTAACCG |
|  | Reverse: GGGCATCACTTCTACCAGGT |
| *Tjp1* | Forward: CTGGTGAAGTCTCGGAAAAATG |
|  | Reverse: CATCTCTTGCTGCCAAACTATC |
| *Ocln* | Forward: CAGGATGCCAATTACCATCAAG |
|  | Reverse: GGGTTCACTCCCATTATGTACA |
| *Cldn1* | Forward: AGATACAGTGCAAAGTCTTCGA |
|  | Reverse: CAGGATGCCAATTACCATCAAG |
| *Muc2* | Forward: CGAGCACATCACCTACCACATCATC |
|  | Reverse: TCCAGAATCCAGCCAGCCAGTC |
| *β-actin* | Forward: GACCTCTATGCCAACACAGT |
|  | Reverse: CACCAATCCACACAGAGTAC |
| *GAPDH* | Forward: TGTTCCTACCCCCAATGTGT |
|  | Reverse: GGTCCTCAGTGTAGCCCAAG |

**Supplementary file 3c. Disease activity index (DAI) parameters and their associated scoring schemes**

| **Score** | **Weight loss (%)** | **Stool consistency** | **Blood in stool** |
| --- | --- | --- | --- |
| 0 | None | Normal | Normal |
| 1 | 1-5 | Slightly loose stool | Small presence of blood |
| 2 | 5-10 | Loose stool | Significant presence of blood |
| 3 | 10-15 | Diarrhea | Gross blood |
| 4 | >15 |  |  |
